# Capacity-expanding O/Cl-bridged catholyte boosts energy density in zero-pressure all-solid-state lithium batteries

**DOI:** 10.1093/nsr/nwaf584

**Published:** 2025-12-26

**Authors:** Houyi Liu, Shuaika Liang, Yuhao Duan, Guanwu Li, Dong Wang, Hongzhang Zhang, Wei Xia, Xiaofei Yang, Xianfeng Li

**Affiliations:** Division of Energy Storage, Dalian Institute of Chemical Physics, Chinese Academy of Sciences, Dalian 116023, China; University of Chinese Academy of Sciences, Beijing 100049, China; Eastern Institute for Advanced Study, Eastern Institute of Technology, Ningbo 315201, China; Division of Energy Storage, Dalian Institute of Chemical Physics, Chinese Academy of Sciences, Dalian 116023, China; University of Chinese Academy of Sciences, Beijing 100049, China; State Key Laboratory of Superhard Materials, and School of Materials Science, and Jilin Provincial International Cooperation Key Laboratory of High-Efficiency Clean Energy Materials, and Electron Microscopy Center, and International Center of Future Science, Jilin University, Changchun 130013, China; State Key Laboratory of Superhard Materials, and School of Materials Science, and Jilin Provincial International Cooperation Key Laboratory of High-Efficiency Clean Energy Materials, and Electron Microscopy Center, and International Center of Future Science, Jilin University, Changchun 130013, China; Division of Energy Storage, Dalian Institute of Chemical Physics, Chinese Academy of Sciences, Dalian 116023, China; Eastern Institute for Advanced Study, Eastern Institute of Technology, Ningbo 315201, China; Division of Energy Storage, Dalian Institute of Chemical Physics, Chinese Academy of Sciences, Dalian 116023, China; Key Laboratory of Long-Duration and Large-Scale Energy Storage, Dalian Institute of Chemical Physics, Chinese Academy of Sciences, Dalian 116023, China; Division of Energy Storage, Dalian Institute of Chemical Physics, Chinese Academy of Sciences, Dalian 116023, China; Key Laboratory of Long-Duration and Large-Scale Energy Storage, Dalian Institute of Chemical Physics, Chinese Academy of Sciences, Dalian 116023, China; Dalian National Laboratory for Clean Energy, iChEM (Collaborative Innovation Center of Chemistry for Energy Materials), Dalian Institute of Chemical Physics, Chinese Academy of Sciences, Dalian 116023, China

**Keywords:** all-solid-state lithium batteries, zero-pressure, capacity-expanding catholyte, polymer-like viscoelasticity, cost-effective

## Abstract

The advancement of all-solid-state lithium batteries (ASSLBs) requires innovative breakthroughs in catholyte design to eliminate the need for external pressure and mitigate the adverse effects of inactive catholytes on energy density. Here, we present a capacity-expanding O/Cl-bridged catholyte (1.2LiOH–FeCl_3_) featuring an abundant, freely rotating Fe_x_O_y_Cl_z_ framework, endowing it with polymer-like viscoelasticity and an impressive ionic conductivity (6.1 mS cm^−1^ at 25°C). The polymer-like viscoelasticity creates a soft interface that alleviates volume changes during cycling, enabling zero-pressure ASSLBs to deliver a high capacity retention of 86.6% after 100 cycles, which is a 35.7% improvement compared to the rigid Li_2_ZrCl_6_ catholyte (50.9%). Moreover, the fast Li^+^ transport capability and variable-valence iron coordination center endow 1.2LiOH–FeCl_3_ catholyte delivering a capacity of 97.7 mAh g^−1^. When used as a catholyte alongside an LiFePO_4_ (LFP) cathode material, it increases capacity by 31.3% (196.4 vs. 149.6 mAh g^−1^_LFP_) and boosts energy density by 21.1% (609.4 vs. 503.4 Wh kg^−1^_LFP_) compared to Li_2_ZrCl_6_ catholyte. Beyond these properties, the 1.2LiOH–FeCl_3_ catholyte offers significant cost advantages, priced at just $2.6 kg^−1^ (16% of the cost of Li_2_ZrCl_6_), and supports scalable production at 60°C, making kilogram- to ton-level manufacturing feasible.

## Teaser

A capacity-expanding O/Cl-bridged catholyte with polymer-like viscoelasticity and high ionic conductivity enables zero-pressure all-solid-state lithium batteries, achieving superior capacity retention and enhanced energy density.

## INTRODUCTION

**Figure 1.**
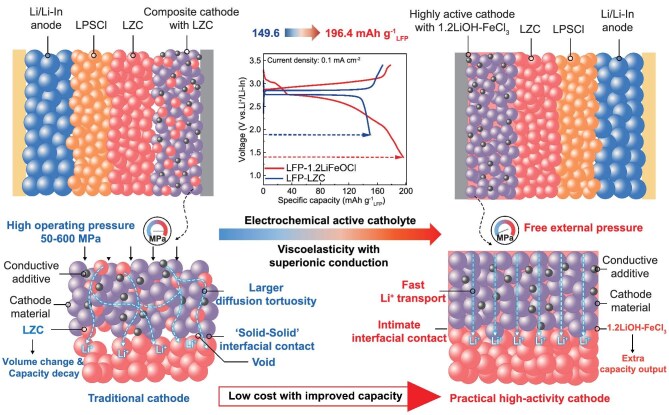
Schematic comparison of the composite cathodes using traditional inert crystalline catholyte (LZC) versus the amorphous capacity-expanding Fe-based oxychloride catholyte (1.2LiOH–FeCl_3_).

All-solid-state lithium batteries (ASSLBs) have garnered intensive attention for their superior safety and higher energy density compared to traditional liquid electrolyte-based lithium-ion batteries, effectively reducing the risk of thermal runaway [1–4]. The performance of ASSLBs, particularly their energy density and cycling life, critically depends on the choice of catholyte and the interface between the cathode active materials (CAMs) and the catholytes [5,6]. In conventional oxide CAMs, such as LiCoO_2_, LiNi_1-x-y_Mn_x_Co_y_O_2_ and LiFePO_4_ (LFP), the integration of high-ionic-conductivity catholytes is necessary to enable efficient lithium-ion (Li⁺) transport [7–9]. Typically, these catholytes constitute 15–30 wt.% of the cathode, providing essential ionic pathways [10]. However, as shown on the left side of Fig. 1 [Li_2_ZrCl_6_ (LZC) is chosen as the representative inactive catholyte], their inactive nature reduces the content of the CAM in the cathode, adversely affecting the energy density of ASSLBs [11,12]. Furthermore, the rigid nature of the CAM–catholyte interface poses another significant challenge. Volume changes during the charging/discharging process will cause the interface to crack, thereby accelerating capacity degradation [13,14]. To address these challenges, there is an urgent need to develop novel catholytes with capacity characteristics and mechanical deformability [15,16].

Recently, halide catholytes with variable-valent metal elements and high ionic conductivity, such as Li_3_TiCl_6_ [17] and Li_3_VCl_6_ [18] have emerged as promising alternatives to inert catholytes, offering extra capacity to improve the energy density of ASSLBs. Nevertheless, their performance still leaves significant room for enhancement, particularly in terms of rate capability, energy density and cost. The limited ionic conductivity (Li_3_TiCl_6_: 1.04 mS cm^−1^; Li_3_VCl_6_: 0.075 mS cm^−1^) and low discharge voltage (Li_3_TiCl_6_: 2.93 V; Li_3_VCl_6_: 2.95 V vs. Li^+^/Li) constrain their applicability. Furthermore, the high cost of raw materials like TiCl_3_ ($1584.9 kg^−1^) and VCl_3_ ($201.4 kg^−1^) poses economic challenges [19]. Additionally, these materials suffer from the limitations of their rigid crystalline structures, which fail to accommodate volume changes during cycling, leading to interface degradation. A high external pressure of 50–600 MPa is required to prolong the cycling life [20]. To address these challenges, the development of deformable capacity-expanding catholytes with higher ionic conductivity, higher discharge voltages and reduced costs is highly recommended. Amorphous solid-state electrolytes (SSEs) offer a promising pathway, exhibiting superior ionic conductivity and deformability compared to their crystalline counterparts [21–23]. For example, amorphous material, such as Li_0.5_AlCl_2.5_O_0.75_, exhibits higher ionic conductivity (1 mS cm^−1^ at 30°C) and viscoelastic properties compared with its crystalline counterpart of LiAlCl_4_, enabling better accommodation of structural changes during cycling. Meanwhile, the Li_0.5_AlCl_2.5_O_0.75_ presents polymer-like viscoelasticity and a low elastic modulus of 1.5 GPa, enabling the successful operation of ASSBs at pressures below 0.1 MPa [24,25]. These characteristics arise from the isotropy of Li^+^ transport in amorphous matrices and their soft, clay-like consistency. Considering cost and electrochemical performance, iron (Fe)-based capacity-expanding catholytes stand out as promising candidates [26]. Fe exhibits higher electronegativity, potentially enabling higher redox voltages [27], while being more economical than Ti [17] and V [18]. Developing amorphous Fe-based capacity-expanding catholytes could combine low cost, high deformability, high ionic conductivity and enhanced energy density, offering an all-in-one solution for next-generation zero-pressure ASSLBs.

Herein, we introduce a capacity-expanding and deformable iron-based oxychloride catholyte (1.2LiOH–FeCl_3_) to enhance the electrochemical performance of zero-pressure ASSLBs. As shown on the right side of Fig. 1, the freely rotating bridging O/Cl endows 1.2LiOH–FeCl_3_ with polymer-like viscoelasticity, which is considered to create a soft and intimate contact interface to enhance cycling stability. Meanwhile, this structural innovation also facilitates rapid Li^+^ transport and exploits the high electronegativity of Fe, delivering an exceptional room-temperature ionic conductivity of 6.1 mS cm^−1^ and a high average discharge voltage of 3.25 V, enabling 1.2LiOH–FeCl_3_ delivering a capacity of 97.7 mAh g^−1^ with 91.4% retention after 300 cycles. When used as a capacity-expanding catholyte alongside an LFP CAM, the zero-pressure ASSLB delivers 196.4 mAh g^−1^_LFP_ and retains 86.6% capacity over 100 cycles—a 31.3% improvement in capacity compared to inactive LZC catholyte with a capacity retention of 50.9%. Economic advantages further amplify its appeal. Priced at just $2.6 kg^−1^, it is significantly cheaper than the widely recognized low-cost LZC catholyte [28] (∼16% of the cost). Additionally, the synthesis process leverages the high reactivity of LiOH and FeCl_3_ under mild conditions (60°C), enabling scalable production at kilogram or ton levels. This study highlights amorphous oxychlorides as a promising class of catholytes for high-energy-density and zero-pressure ASSLBs, warranting further investigation.

## RESULTS AND DISCUSSION

### Synthesis and properties of xLiOH–FeCl_3_ catholyte

**Figure 2.**
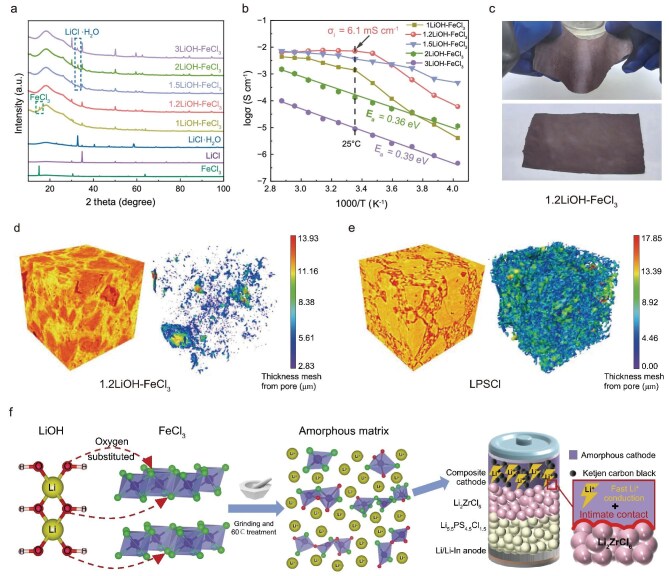
Synthesis and properties of the xLiOH–FeCl_3_ catholytes. (a) XRD patterns of xLiOH–FeCl_3_ (x = 1–3), FeCl_3_, LiCl and LiCl‧H_2_O. (b) Arrhenius plots of xLiOH–FeCl_3_ materials (x = 1–3). (c) The bending membrane of 1.2LiOH–FeCl_3_ was obtained by the rolling process. 3D volume rendered images and a mesh 3D model from micro-level XCT of (d) 1.2LiOH–FeCl_3_ and (e) LPSCl pellets achieved under a cold-pressure of 125 MPa. In the mesh 3D model, colors represent the cracks and voids. (f) Schematic diagram of the oxygen-bridging reaction and ASSLB configuration.

The Fe-based oxychloride catholytes are synthesized by a cost-effective oxygen-bridging reaction conducted at a mild temperature of 60°C, with LiOH and FeCl_3_ as the starting materials. The molar ratio of LiOH to FeCl_3_ is varied (x = 1–3) to produce a series of materials, denoted as xLiOH–FeCl_3_. As shown in Fig. S1, with the ratio of LiOH decreasing, the xLiOH–FeCl_3_ gradually changes from a powder state (x = 3, 2) to a clay-like state (x = 1.5, 1.2). Structural evolution is investigated using X-ray diffraction (XRD), revealing that when x = 1, unreacted FeCl_3_ is detected (Fig. 2a). At x = 1.2, the FeCl_3_ peaks diminish, indicating a completed reaction, while faint crystalline LiCl signals appear, confirming the formation of an amorphous primary structure. With higher LiOH content (x > 1.2), new peaks at 32.8° and 33.1° belonging to LiCl‧H_2_O emerge, reducing the ionic conductivity due to the formation of non-conductive phases. Among the series, 1.2LiOH–FeCl_3_ exhibits the highest room-temperature ionic conductivity of 6.1 mS cm^−1^ (Fig. 2b). Meanwhile, Arrhenius plots reveal distinct polymer-like behavior for the xLiOH–FeCl_3_ (x = 1.2, 1.5) samples with an inflection point at 15°C, correlating with their clay-like states and enhanced deformability. Similar to other ‘polymer-like’ inorganic glass electrolytes [24,30], the non-Arrhenius ionic conductivity of 1.2LiOH–FeCl_3_ results from its temperature-dependent viscosity, a behavior underpinned by its excellent rheological properties. The polymer-like behavior is further proved by the bending membrane of 1.2LiOH–FeCl_3_ obtained by the rolling process in Fig. 2c. In contrast, powder samples (x = 2, 3) exhibit a linear temperature–conductivity relationship, with activation energies of 0.36 and 0.39 eV, respectively, resembling typical crystalline [21] and halide catholytes [17,18]. Furthermore, rheometer measurements of the 1.2LiOH–FeCl_3_ catholyte reveal a dominant elastic response, characterized by storage (G′) and loss (G″) moduli values of 10^3^–10^5^ Pa and a phase shift (δ) of <45° across the measured frequency range (Fig. S2). This behavior is indicative of clay-like solid properties [31].

For ASSLBs, maintaining a low stack pressure is essential during manufacturing to ensure performance and reliability, while exploring soft and compressible catholytes is a promising strategy to address these requirements [30,31]. The optical image of a 1.2LiOH–FeCl_3_ pellet under a pressure of 125 MPa is shown in Fig. S3, where LZC and Li_5.5_PS_4.5_Cl_1.5_ (LPSCl) are chosen for comparison. Generally, all three samples form complete pellets under cold pressing, but significant differences emerge in scanning electron microscopy (SEM) images. The SEM images in Figs S4 and S5 reveal that 1.2LiOH–FeCl_3_ forms a dense structure without noticeable pores, while both LPSCl and LZC SSEs exhibit substantial porosity. To quantify this difference, X-ray computed tomography (XCT) is employed to analyze pore distribution. The 3D mesh model of the cold-pressed 1.2LiOH–FeCl_3_ pellet (Fig. 2d and Fig. S6) displays a tightly interconnected structure with minimal voids or gaps, yielding a calculated porosity of just 1.03%. In contrast, XCT analysis of the LPSCl pellet (Fig. 2e and Fig. S7) reveals numerous voids, with a calculated porosity of 16.46%. The XCT findings align with the SEM observations and emphasize the superior deformability of the clay-like 1.2LiOH–FeCl_3_ material. Considering its high ionic conductivity and deformability, and the versatile valence states of Fe^3+^, 1.2LiOH–FeCl_3_ is selected for detailed electrochemical evaluation in ASSLBs, which is expected to facilitate a well-contacted interface, accommodating volume changes and prolonging the cycling life of ASSLBs (Fig. 2f).

### Structure characterization and large-scale production of representative 1.2LiOH–FeCl_3_ catholyte

**Figure 3.**
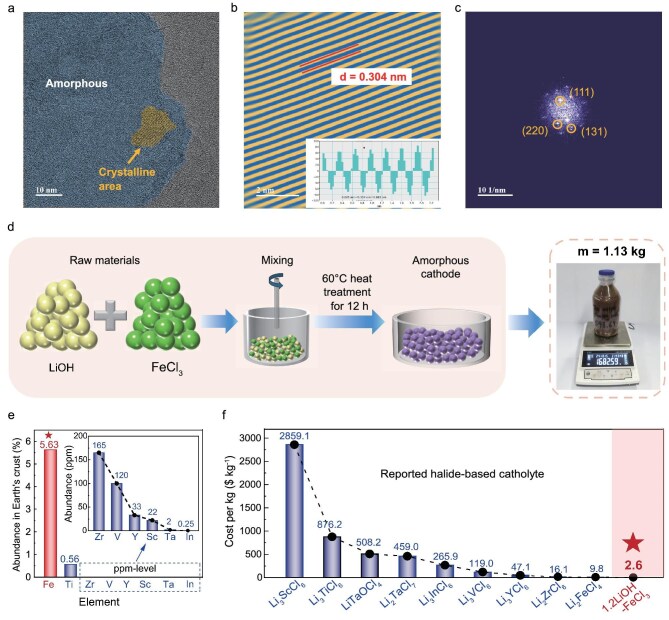
Corresponding structure characterization and large-scale production demonstration of representative 1.2LiOH–FeCl_3_ catholyte. (a) Cryo-TEM images of the 1.2LiOH–FeCl_3_ material. (b) The lattice fringe of the crystalline phase. (c) The fast Fourier transform of a crystalline lattice fringe. (d) Schematic diagram of scaled-up 1.2LiOH–FeCl_3_ cathode synthesis route (1.13 kg in the bottle). (e) Abundance of Fe, Ti and V in the Earth’s crust. (f) Estimated unit prices of several reported halide-based catholytes [9,17,18,27,28,32–35].

The XRD analysis reveals that 1.2LiOH–FeCl_3_ is composed of a combination of an amorphous phase and nanocrystalline LiCl. To gain nanostructure insights, cryogenic transmission electron microscopy (cryo-TEM) is performed. As depicted in Fig. 3a–c, the selected crystalline region displays a lattice fringe with measured spacings of 0.304 nm, corresponding to the (111) plane of cubic LiCl nanocrystals. Together, the XRD and cryo-TEM findings confirm that 1.2LiOH–FeCl_3_ consists of both an amorphous phase and nanocrystalline LiCl. To quantitatively determine the LiCl content in the 1.2LiOH–FeCl_3_, an internal standard method is employed. The analysis confirms a nanocrystalline LiCl content of 12 wt.%. (Fig. S8). Considering nanocrystalline LiCl is a non-ionic conductor, the high ionic conductivity of 1.2LiOH–FeCl_3_ can be attributed to the amorphous phase, which facilitates isotropic Li^+^ migration and exhibits high deformability [36].

In terms of scalability and cost, the 1.2LiOH–FeCl_3_ material offers significant advantages. The synthesis process of the 1.2LiOH–FeCl_3_ catholyte involves a simple one-pot oxygen-bridging reaction at 60°C, enabling kilogram-level production in a laboratory, as demonstrated by the synthesis of 1.13 kg of 1.2LiOH–FeCl_3_ (Fig. 3d and Fig. S9). Furthermore, to assess the scalability of chloride SSEs and catholytes, the cost assessment for constituent materials is conducted as follows: prices for commercially available commodities such as LiOH, FeCl_3_ and LiCl are sourced from supplier websites, while the costs of all other materials are estimated according to the methodology of Hart *et al.* to reflect projected commercial-scale production. (Fig. S10, Tables S1 and S2) [19,28]. The abundance of iron (5.63% in the Earth’s crust) and the low cost of FeCl_3_ ($0.4 kg^−1^) contribute to an ultralow synthesis cost of 1.2LiOH–FeCl_3_, estimated at $2.6 kg^−1^ (Fig. 3e and f). This contrasts sharply with other reported halide-based catholytes such as Li_3_ScCl_6_ [24] ($2859.1 kg^−1^), Li_3_TiCl_6_ [17] ($876.2 kg^−1^), LiTaOCl_4_ [26] ($508.2 kg^−1^), Li_3_VCl_6_ [18] ($119.0 kg^−1^), Li_2_ZrCl_6_ [29] ($16.1 kg^−1^) and Li_2_FeCl_4_ [19] ($9.8 kg^−1^), etc. These features underscore its strong potential for future commercialization in ASSLBs [37,38].

### Structural analysis of amorphous 1.2LiOH–FeCl_3_ catholyte

**Figure 4.**
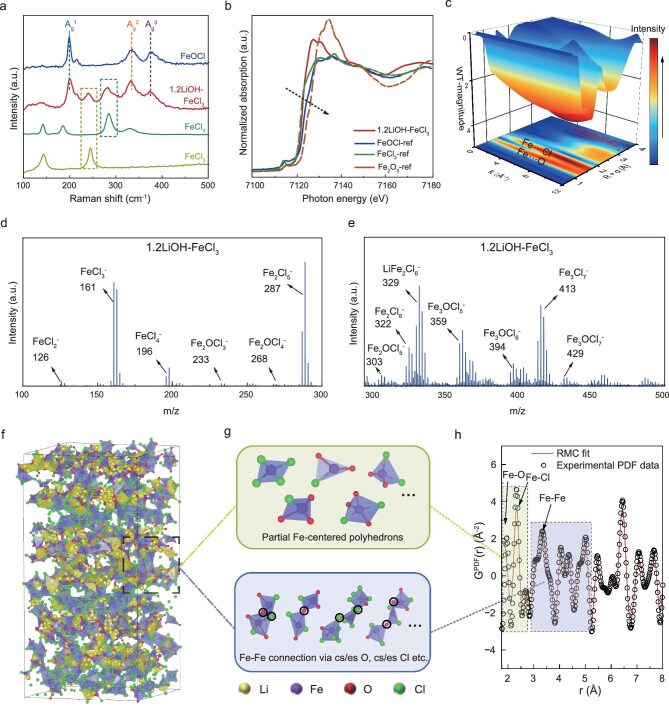
The structural analysis of amorphous 1.2LiOH–FeCl_3_ catholyte. (a) Raman spectra of the 1.2LiOH–FeCl_3_, and reference FeCl_2_, FeCl_3_ and FeOCl. (b) Fe K-edge XAS of referential FeOCl, FeCl_3_, Fe_2_O_3_ and 1.2LiOH–FeCl_3_. (c) WTs for the k^3^-weighted Fe K-edge EXAFS signals of 1.2LiOH–FeCl_3_. (d and e) Negative-ion TOF-SIMS of 1.2LiOH–FeCl_3_ pellet. (f) Atom arrangement in the modeled 1.2LiOH–FeCl_3_ supercell. (g) Typical Fe-centered building blocks, edge-sharing (es) and corner-sharing (cs) Fe-centered polyhedral in the 1.2LiOH–FeCl_3_ supercell model. (h) RMC fits the experimental G(r) of 1.2LiOH–FeCl_3_.

To clarify the relationship between structure and deformability, Raman spectroscopy is used to analyze the Li^+^ transport environment and structural properties of the representative 1.2LiOH–FeCl_3_ material. As shown in Fig. 4a, 1.2LiOH–FeCl_3_ displays five characteristic peaks at 198.6, 244.5, 284.2, 334.5 and 379.7 cm^−1^. Three peaks at 198.6, 244.5 and 379.7 cm^−1^, similar to those of FeOCl [39], correspond to the A_g_^1^, A_g_^2^ and A_g_^3^ vibrational modes. The A_g_^1^ and A_g_^2^ modes represent out-of-plane Fe–O bond vibrations, while the A_g_^3^ mode reflects Fe–Cl bond stretching [37]. Moreover, the two peaks at 244.5 and 284.2 cm^−1^ align with the crystal lattice vibrations of FeCl_3_ and FeCl_2_, respectively. These observations confirm the coexistence of structurally distinct coordination environments within the amorphous phase, suggesting a heterogeneous local structure.

To further explore the atomic-scale environment, Fe K-edge X-ray absorption near-edge structure (XANES) and extended X-ray absorption fine structure (EXAFS) analyses are conducted. As shown in Fig. 4b, the absorption edge energy (E_0_) of 1.2LiOH–FeCl_3_ is between that of FeOCl and FeCl_3_. This indicates an increased oxidation state of Fe ions in FeCl_3_, likely due to the substitution of Cl with a higher electronegativity O, reducing the electron density around Fe^3+^. Phase-uncorrected radial distribution functions (RDFs) derived from wavelet-transformed (WT) EXAFS reveal Fe–O and Fe–Cl coordination distances of 1.4 and 1.7 Å, respectively, consistent with FeOCl-like structures (Fig. 4c, Figs S11–S13 and Tables S3 and S4). Different from the RDF result of the FeOCl reference, the signal intensity of the Fe–Cl peak in 1.2LiOH–FeCl_3_ is significantly stronger than that of Fe–O, indicating more Fe–Cl coordination structures in the amorphous phase.

Time-of-flight secondary ion mass spectrometry (TOF-SIMS) is further employed to identify the coordination structure of 1.2LiOH–FeCl_3_ (Fig. 4d and e). Signals assigned to Fe_x_Cl_y_ clusters (e.g. FeCl_3_^−^, FeCl_4_^−^, Fe_2_Cl_6_^−^, Fe_3_Cl_7_^−^ etc) and Fe_x_O_y_Cl_z_ iron–base oxyhalide polyanions (e.g. Fe_2_OCl_3_^−^, Fe_2_OCl_4_^−^, Fe_3_OCl_5_^−^ etc) are detected, which agrees with the Raman and EXAFS results. These findings indicate the presence of two primary structural units, Fe_x_Cl_y_ and Fe_x_O_y_Cl_z_, within the amorphous phase. The formation of these structural motifs is attributed to bridging oxygen atoms from LiOH coordinating with Fe centers in FeCl_3_. This interaction disrupts the original layered FeCl_3_ structure, leading to the emergence of a disordered amorphous network.

Further structural insights are derived from *ab initio* molecular dynamics (AIMD) simulations of 1.2LiOH–FeCl_3_. Starting from the refined crystal structure of LiFe_2_O_2_Cl_2_ (Fig. S14), identified to have the lowest Coulomb energy (Fig. S15), an amorphous model of 1.2LiOH–FeCl_3_ is generated (Fig. S16). To better understand the amorphous structure of 1.2LiOH–FeCl_3_, reverse Monte Carlo (RMC) modeling based on total scattering data is conducted [14,33]. As shown in Fig. 4h, the RMC-derived generalized pair distribution function [G^PDF^(r)] provides a statistical representation of the local structural configurations (Fig. 4f). The extracted statistical information around Fe atoms reveals that Fe_x_Cl_y_ and Fe_x_O_y_Cl_z_ units serve as the primary Fe-centered building blocks, and polyhedral units are predominantly corner-sharing via Cl or O atoms (Fig. 4g). Analogous to the motion of polymer backbones, the rotation and bending of the corner-sharing network generate free volume, imparting the 1.2LiOH–FeCl_3_ with high deformability [16,40]. These structural features provide a comprehensive explanation for the exceptional deformability of the amorphous 1.2LiOH–FeCl_3_ material [22–24,41].

### Electrochemical performance of assembled ASSLBs

Based on the aforementioned characteristics, 1.2LiOH–FeCl_3_ material is first employed as a CAM to investigate the charge–discharge behavior in ASSLBs. The cell adopts a dual-layer SSE configuration, with LZC SSE positioned adjacent to the cathode and LPSCl SSE stabilizing the Li or Li–In anodes [26].

**Figure 5.**
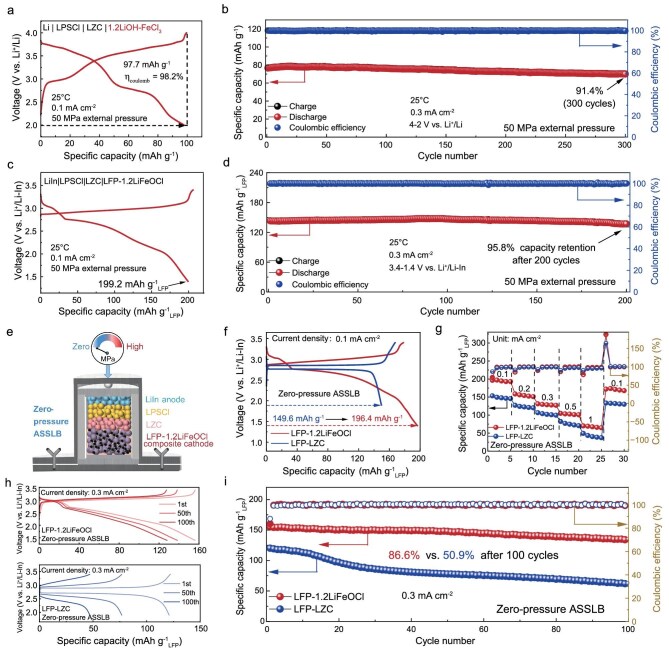
Electrochemical performance of ASSLBs. (a) Charge/discharge profiles at 0.1 mA cm^−2^ and (b) cycling performance at 0.3 mA cm^−2^ of 1.2LiOH–FeCl_3_ CAM under an external pressure of 50 MPa. (c) Charge/discharge profiles at 0.1 mA cm^−2^ and (d) cycling performance at 0.3 mA cm^−2^ of LFP–1.2LiFeOCl composite cathode under an external pressure of 50 MPa. (e) Schematic diagram of the zero-pressure ASSLB configuration. (f) Charge/discharge profiles of LFP–1.2LiFeOCl and LFP–LZC composite cathodes at 0.1 mA cm^−2^. (g) The rate performance of LFP–1.2LiFeOCl and LFP–LZC composite cathodes. (h) Charge/discharge profiles and (i) cycling stability of LFP–1.2LiFeOCl and LFP–LZC composite cathodes at 0.3 mA cm^−2^.

At a current density of 0.1 mA cm^−2^, the catholyte-free ASSLB containing 95 wt.% 1.2LiOH–FeCl_3_ exhibits two distinct discharge plateaus at 3.7 V and 2.8 V, delivering a specific capacity of 97.7 mAh g^−1^ (Fig. 5a). Cyclic voltammetry (CV) further confirms the presence of two reversible redox couples at 3.7/3.65 and 3.0/2.75 V (Fig. S17), which can be assigned to Fe^2+^/Fe^3+^ redox chemistry coupled with the Li^+^ intercalation/deintercalation reactions in the coordination environment of Fe_x_Cl_y_ and Fe_x_O_y_Cl_z_, which is supported by the discharging profiles of FeCl_3_ and FeOCl CAMs in Fig. S18. To elucidate the discharging reaction mechanism, *ex situ* X-ray absorption spectroscopy (XAS) measurement is performed on the 1.2LiOH–FeCl_3_ cathode at different states of discharge (Fig. S19). The Fe K-edge XANES spectra (Fig. S19a) shift toward lower energies during discharge, indicating a reduction of Fe^3+^ to Fe^2+^ [26]. This change in oxidation state is further supported by the Fe K-edge EXAFS results (Fig. S19b), which reveal structural evolution in the local coordination environment. At the end of the first discharge plateau (down to 2.86 V), the average Fe–Cl bond length increases from 1.91 to 2.02 Å, while the Fe–O distance remains unchanged. This elongation of the Fe–Cl bond is primarily due to the Li^+^ intercalation reaction in the coordination environment of Fe_x_Cl_y_ anionic framework. The Coulombic attraction between Li^+^ and the surrounding Cl^−^ ions weakens and stretches the original Fe–Cl bonds [42]. The corresponding discharge plateau matches that of the FeCl_3_ cathode, supporting the idea that Li⁺ first intercalates into the chloride-rich framework. As the discharge continues (down to 2.00 V), the Fe–O bond elongates from 1.39 to 1.54 Å, accompanied by further expansion of the Fe–Cl distance. This later-stage Fe–O bond lengthening arises from the subsequent insertion of Li^+^ into the coordination environment of the Fe_x_O_y_Cl_z_ framework. The increasing Li^+^ content introduces electrostatic and steric effects that push oxygen ligands away from Fe centers, thereby extending Fe–O bonds. The corresponding voltage plateau in this stage aligns with that of the FeOCl cathode, confirming the insertion of Li^+^ into the oxychloride structure. The remarkable capacity retention (91.4% after 300 cycles at 0.3 mA cm^−2^ in Fig. 5b) and nearly identical CV profiles confirm the intimate interfacial contact and the highly reversible Fe^2+^/Fe^3+^ redox chemistry, endowing it with the capability to be a capacity-expanding catholyte [43].

To evaluate the electrochemical performance of 1.2LiOH–FeCl_3_ as an active catholyte, LFP is selected as the CAM due to its analogous electrochemical reaction process and compatible voltage window. As shown in Fig. 5c, a composite cathode comprising LFP, 1.2LiOH–FeCl_3_, and carbon (denoted as LFP–1.2LiFeOCl) exhibits two distinct discharge plateaus and delivers a high discharge capacity of 199.2 mAh g^−1^_LFP_ (based on the mass of LFP) under an external pressure of 50 MPa. It exceeds the theoretical discharge capacity of LFP (170 mAh g^−1^), which can be attributed to additional capacity contributed by the 1.2LiOH–FeCl_3_ catholyte. Meanwhile, a high capacity retention of 95.8% for 200 cycles at 0.3 mA cm^−2^ is achieved by the LFP–1.2LiFeOCl cathode, indicating the high compatibility between LFP and 1.2LiOH–FeCl_3_ (Fig. 5d). To advance the practical application of ASSLBs, it is critical to minimize the stack pressure required during electrochemical cycling [20]. Therefore, the ASSLB measurements are further conducted under zero-pressure using LFP–1.2LiFeOCl composite cathode (Fig. 5e). As shown in Fig. 5f, the zero-pressure ASSLB delivers a high discharge capacity/energy density of 196.4 mAh g^−1^_LFP_/609.4 Wh kg^−1^_LFP_ vs. Li^+^/Li. In comparison, a reference LFP–LZC composite cathode displays only the characteristic LFP plateau with a lower capacity/energy density of 149.6 mAh g^−1^_LFP_/503.4 Wh kg^−1^_LFP_ [44]. In other words, the integration of 1.2LiOH–FeCl_3_ significantly enhances the capacity and energy density by 31.3% and 21.1%, respectively (Fig. S20). Moreover, as shown in Fig. 5g–i and Fig. S21, the zero-pressure ASSLB utilizing the LFP–1.2LiFeOCl cathode maintains 86.6% capacity retention after 100 cycles at 0.3 mA cm^−2^ and delivers 67.6 mAh g^−1^_LFP_ at a high rate of 1 mA cm^−2^ (3.3 C), which is superior to that of LFP–LZC cathode (50.9% capacity retention after 100 cycles and 40.5 mAh g^−1^_LFP_ at 1 mA cm^−2^). The improved capacity retention and rate capability highlight the positive effect of the high-ionic-conductivity 1.2LiOH–FeCl_3_ on accommodating volume change and expanding capacity during cycling. To understand the interfacial evolution during cycling, post-cycling electrochemical impedance spectroscopy (EIS) and SEM are carried out. The post-cycling EIS reveals a much smaller increase in interfacial impedance for the LFP–1.2LiFeOCl cathode (+133 Ω) compared to the LFP–LZC cathode (+260 Ω, Fig. S22) after 100 cycles. The findings point to the superior ability of 1.2LiOH–FeCl_3_ in stabilizing the electrode–electrolyte interface during cycling. It is further supported by the cross-sectional SEM images. As shown in Fig. S23, the LFP–1.2LiFeOCl cathode maintains a void-free interface after cycling, which can be attributed to the soft 1.2LiOH–FeCl_3_ on accommodating volume change, resulting in enhanced capacity retention and reduced interfacial resistance. In contrast, the rigid ‘solid–solid’ contact in the LFP–LZC cathode leads to extensive void formation and interfacial cracking, ultimately degrading electrochemical performance [45].

### Dynamic interfacial evolution in zero-pressure ASSLBs

**Figure 6.**
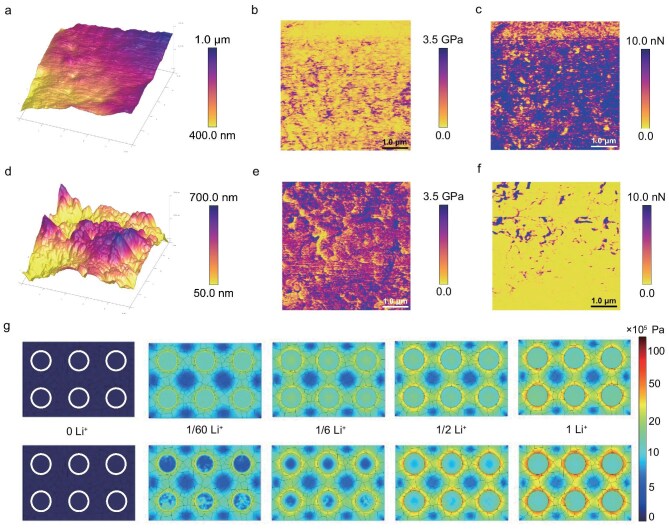
Dynamic interfacial evolution of composite cathodes in zero-pressure ASSLBs. (a–c) AFM images: (a) upper surface morphology, (b) Young’s modulus and (c) adhesion of 1.2LiOH–FeCl_3_ pellet. (d–f) AFM images: (d) upper surface morphology, (e) Young’s modulus and (f) adhesion of LZC pellet. (g) Stress distribution inside composite cathodes of LFP–1.2LiFeOCl (top) and LFP–LZC (bottom) during discharging/lithiation. The white circles represent the initial LFP particles, and the irregular polygons represent catholytes. 0 Li^+^ and other labels represent different discharge states of composite cathodes.

To elucidate the interfacial failure mechanisms in zero-pressure ASSLBs, dynamic monitoring of interfacial evolution is conducted. Atomic force microscopy (AFM) is employed to investigate the mechanical properties of 1.2LiOH–FeCl_3_ and LZC catholytes [46]. As displayed in Fig. 6a and d, compared to the uneven height variations observed in the LZC pellet, the 1.2LiOH–FeCl_3_ pellet exhibits a smoother surface morphology with no detectable voids, corroborating observations from SEM and XCT. The difference in morphology can be attributed to the higher deformability of 1.2LiOH–FeCl_3_ under pressure. It is further confirmed by Young’s modulus testing in Fig. 6b and e, and Fig. S24. As observed, 1.2LiOH–FeCl_3_ displays a lower Young’s modulus of ∼1.2 GPa, which is roughly one-third of that of LZC (∼3.3 GPa), indicating greater softness and deformability. Additionally, 1.2LiOH–FeCl_3_ shows stronger adhesion to the AFM tip than powdery LZC (Fig. 6c and f), further supporting its suitability for zero-pressure ASSLB architectures.

Using the mechanical parameters listed in Table S5, a finite element simulation is performed to dynamically reconstruct the interfacial reaction process, enabling real-time visualization of interface evolution [47]. During discharge (lithiation), the LFP particles in the LFP–1.2LiFeOCl cathode exhibit a higher degree of lithiation compared to those in the LFP-LZC cathode (Fig. S25). This enhancement is attributed to the superior ionic conductivity and faster electrochemical kinetics of 1.2LiOH–FeCl_3_ (Fig. S26), which promotes more efficient ion exchange at the interface (0.094 vs. 0.015 mA cm^−2^ for LFP–LZC electrode) and leads to improved rate performance. Furthermore, mechanical stress analysis reveals significant stress accumulation (σ_1Li+_ = 16 MPa) between the powdery LFP and LZC particles in the LFP-LZC cathode after lithiation (Fig. 6g). In contrast, the exceptional deformability and viscoelasticity of 1.2LiOH–FeCl_3_ effectively mitigate interfacial stress in the LFP-1.2LiFeOCl cathode (σ_1Li+_ = 5.7 MPa, which is roughly one-third of that of the interfacial stress in the LFP–LZC composite cathode), providing a more stable mechanical environment. These findings are consistent with the higher capacity retention and lower interfacial impedance in LFP–1.2LiFeOCl, which is the key to the improved cycling stability in the zero-pressure ASSLBs by accommodating the volume change during cycling [48]. These results validate 1.2LiOH–FeCl_3_ as an effective capacity-expanding catholyte, supporting the development of advanced zero-pressure ASSLBs with improved electrochemical performance and enhanced economic viability.

## CONCLUSION

In summary, we developed a superionic conductive halide catholyte with polymer-like viscoelasticity to solve the stiff ‘solid–solid’ interface and reduce the contribution of inactive catholyte to worsening the energy density. As-prepared 1.2LiOH–FeCl_3_ material possesses an abundant freely rotating O/Cl-bridged Fe_x_O_y_Cl_z_ framework, endowing it with exceptional deformability to create a soft interface and an impressive ionic conductivity (6.1 mS cm^−1^ at 25°C). The superionic conductivity and variable-valence iron coordination center enable the 1.2LiOH–FeCl_3_ material to be a capacity-expanding catholyte. The composite cathode consisting of LiFePO_4_ and 1.2LiOH–FeCl_3_ displays an impressive capacity/energy density of 196.4 mAh g^−1^_LFP/_609.4 Wh kg^−1^_LFP_, improving the capacity and energy density by 31.3% and 21.1% compared to LZC catholyte, respectively. Meanwhile, the high polymer-like viscoelasticity of the 1.2LiOH–FeCl_3_ catholyte helps accommodate the volume change during cycling, enabling the zero-pressure ASSLBs to deliver a high capacity retention of 86.6% after 100 cycles, which is a 35.7% improvement compared to the rigid LZC catholyte. Exceeding the superionic conductivity and high deformability, the 1.2LiOH–FeCl_3_ material exhibits two key characteristics necessary for industrialization: low cost and mass production capability. It presents a low cost of $2.6 kg^−1^ (16% of LZC SSE) and kg-, even ton-level production capability (produced at 60°C). These findings underscore the potential of 1.2LiOH–FeCl_3_ as a highly promising active catholyte, combining electrochemical stability and multifunctionality to significantly enhance ASSLB performance.

## METHODS

The experimental details can be found in the online Supplementary data.

## Supplementary Material

nwaf584_Supplemental_File
