# Supplementary material for: Capacity-expanding O/Cl-bridged catholyte boosts energy density in zero-pressure all-solid-state lithium batteries
Source: Natl Sci Rev. 2025 Dec 26;13(4):nwaf584. doi: 10.1093/nsr/nwaf584 (PMC12875118; doi:10.1093/nsr/nwaf584)
Supplement: nwaf584_Supplemental_File [file nwaf584_supplemental_file.pdf]

Supporting Information

Capacity-Expanding O/Cl-bridged Catholyte Boosts Energy Density in Zero-Pressure All-Solid-State Lithium Batteries

Houyi Liu[1,2], Shuaika Liang[3], Yuhao Duan[1,2], Guanwu Li[4], Dong Wang[4], Hongzhang Zhang[1], Wei Xia[3,*], Xiaofei Yang[1,5,*], Xianfeng Li[1,5,6,*]

[1]Division of Energy Storage, Dalian Institute of Chemical Physics, Chinese Academy of Sciences, Dalian 116023, China;

[2]University of Chinese Academy of Sciences, Beijing 100049, China;

[3]Eastern Institute for Advanced Study, Eastern Institute of Technology, Ningbo 315201, China;

[4]State Key Laboratory of Superhard Materials, and School of Materials Science, and Jilin Provincial International Cooperation Key Laboratory of High-Efficiency Clean Energy Materials, and Electron Microscopy Center, and International Center of Future Science, Jilin University, Changchun, 130013, China;

[5]Key Laboratory of Long-Duration and Large-Scale Energy Storage, Dalian Institute of Chemical Physics, Chinese Academy of Sciences, Dalian 116023, China.

[6]Dalian National Laboratory for Clean Energy, *i*ChEM (Collaborative Innovation Center of Chemistry for Energy Materials), Dalian Institute of Chemical Physics, Chinese Academy of Sciences, Dalian 116023, China.

***Corresponding authors.** E-mails: yangxf@dicp.ac.cn; lixianfeng@dicp.ac.cn; wxia@eitech.edu.cn

Experimental section

Synthesis methods:

LiOH (99.9%, anhydrous, Aladdin) and $FeCl_3$ (99.9%, anhydrous, Aladdin) were used as received. The xLiOH-$FeCl_3$ materials were synthesized by an oxygen-bridging reaction. Firstly, stoichiometric amounts of LiOH and $FeCl_3$ were mixed by hand in an agate mortar. Subsequently, the mixture was heated at 60°C for 12 h in an Ar atmosphere. After cooling down, the obtained products are labeled as xLiOH-$FeCl_3$. The Li_2ZrCl_6 solid-state electrolyte (SSE) with a room-temperature (RT) ionic conductivity of 0.69 mS cm^{-1} was synthesized by a ball-milling method according to the previous report.[1] Commercial $Li_{5.5}PS_{4.5}Cl_{1.5}$ SSE (China Automotive Battery Research Institute Co.) with an RT-ionic conductivity of 4.8 mS cm^{-1} was used directly as the SSE stabilizing the Li anode.

Characterization:

Powder X-ray diffraction (XRD) was collected on a Rigaku Ultima IV diffractometer with Cu Kα radiation (λ = 1.54178 Å) with a Kapton film to avoid air exposure. X-ray computed tomography (XCT) was performed at room temperature using a ZEISS Xradia 515 Versa 3D X-ray microscope. The morphology was obtained with a field-emission scanning electron microscope (SEM, JEOL JSM-7800F). The crystal structure was characterized by an environmental transmission electron microscope (E-TEM, Quanta 650 FEG). Raman spectra were acquired using a Bruker Optics Senterra Raman spectrophotometer equipped with a 532.4 nm laser. Fe K-edge X-ray absorption spectra (XAS) were performed was conducted at beamline 20U of the Shanghai Synchrotron Radiation Facility (SSRF), China, where the samples were mixed with BN and pressed into pellets. All XAS data were analyzed with the Athena software, and the extended X-ray absorption fine structure (EXAFS) data were processed with the Artemis program. X-ray atomic pair distribution function (PDF) was performed at beamline ID31 at the European Synchrotron Radiation Facility (ESRF). The ionic conductivity of as-prepared

xLiOH-$FeCl_3$ materials was tested by electrochemical impedance spectroscopy (EIS) measurement using model cells with controlled temperatures from -25 to 75 °C. Firstly, 120 mg xLiOH-$FeCl_3$ in the model cells were placed between two stainless steel rods and then pressed into a pellet under a pressure of 60 MPa (diameter 10 mm). The EIS was collected on a multichannel VMP3 potentiostat/galvanostat (Bio-logic) in the frequency range of 1 Hz ~ 7 MHz and the voltage amplitude of 100 mV. TOF-SIMS measurement was performed on the TOF.SIMS 5 instrument (ION-TOF GmbH, Germany) in negative ion mode. The sputtering using a Cs^+ ion beam with an acceleration voltage of 1 kV on an area of 500 μm×500 μm. The analysis beam was Bi^{3+} ion with an acceleration voltage of 30 kV and a flood gun was used for charge neutralization. The sample was transferred from the glovebox to the module via a holder under Ar atmosphere. Data processing was performed by the SurfaceLab software. AFM (Bruker Dimension ico) measurement was performed in the glovebox under an Ar atmosphere. Data processing was performed by the NanoScope Analysis software. Fullproof software was used to do the Rietveld refinement for the lab-XRD (5° min^{-1}). Internal standard method was employed to quantify the amorphous content in all samples. Li_2S was used as the internal standard (50 wt.%), which was mixed with each interested sample by hand grinding using an agater mortar. Dynamic oscillatory measurements were performed using a rheometer (HAAKE MARS 60, Thermo Scientific, USA) equipped with a cone-and-plate geometry (2° cone angle, 40 mm diameter, 0.5~1.5 mm testing gap). The frequency was swept from 1.0 to 50 Hz with a 0.5% strain amplitude at 25 °C.

Electrochemical characterizations:

For the ASSLBs using 1.2LiOH-$FeCl_3$ cathode, a composite cathode was prepared by manually mixing the 1.2LiOH-$FeCl_3$ sample and KB 600 in an agate mortar at a weight ratio of 95:5 for 0.5 hours. For the zero-pressure ASSLBs using $LiFePO_4$-1.2LiOH-$FeCl_3$ (LFP-1.2LiFeOCl) or LFP-LZC cathode, a composite cathode was prepared by

ball-milling LFP, 1.2LiOH-$FeCl_3$ (or LZC), and KB600 at a weight ratio of LFP/1.2LiOH-$FeCl_3$ (or LZC)/KB = 65/32/3 under 180 r/min for 2 hours. For the battery assembly, 50 mg of LZC and 50 mg of LPSCl SSEs were subsequently loaded into the model cell (Beijing Zhongke Wanyuan Technology Co. Ltd.) and cold-pressed at 125 MPa. Afterward, 4 mg cm^{-2} (3.2 mg) of the cathode composite was uniformly spread onto the surface of the compressed LZC side with a pressure of 500 MPa, and a 10 μm Al foil was introduced as the cathode current collector. Lastly, a piece of 50 μm Li foil (or 100 μm Li-In) anode was added to the LPSC side and a 10 μm Cu foil was introduced as the anode current collector. All the processes were conducted in an Ar-filled glove box. The galvanostatic charge-discharge tests were carried out using a LAND CT-2001A system with an operating voltage of 2~4 V vs. Li^+/Li (or 1.4~3.4 V vs. Li^+/Li-In) under various current densities ranging from 0.1 to 2 mA cm^{-2} at 25 °C. Cyclic voltammetry (CV) measurements were conducted on a multichannel VMP3 potentiostat/galvanostat (Bio-logic) with an operating voltage of 2~4 V vs. Li^+/Li (or 1.4~3.4 V vs. Li^+/Li-In) at a scan rate of 0.1 mV s^{-1}.

Cost analysis:

The prices of commodities can be checked directly on the website (LiOH, LiCl, and $FeCl_3$). The prices of the chemicals in bulk purchase were extrapolated from laboratory-scale prices using the methodology proposed by Hart et al.[2]. This method is based on the premise that the unit price, P, and the purchase quantity, Q, of the chemicals adhere to this general relationship:

$$log_{10}P = log_{10}a + b\times log_{10}Q \quad (1)$$

where a and b are constants for a given chemical. Therefore, by using a series of unit prices (P) and quantities (Q) for laboratory-scale purchases, the unit price for bulk quantity purchases can be estimated through linear extrapolation using Eq. (1). The reliability of this estimation is reflected by the absolute value of the linear correlation coefficient, |r|, between $log_{10}P$ and $log_{10}Q$ (a value closer to unity indicates higher reliability). The laboratory-scale prices used for this estimation are mainly from Aladdin's official website, except for $TiCl_3$ and VCl_3, which are sourced from Alfa

Aesar [3] and Adamas-bata [4]. Additional details about these chemicals, including stock number, purchase quantity, and purity, can be found in Tables S1 and S2.[1, 4]

Computational methods:

First-principles DFT calculations were carried out using the spin-polarized GGA+U [5] method with the Vienna Ab initio Simulation Package (VASP) [6]. Geometric optimization calculations consistent with those Material Project (MP) [7] were performed with a 3 × 1 × 3 Monkhorst-Pack [8] k-point grid with an energy cut-off of 520 eV for plane waves. Projector augmented wave (PAW) [9, 10] method was used for core-valence interactions. Ab initio molecular dynamics (AIMD) simulations were performed under the GGA with NPT ensemble using a 2fs time step to obtain an amorphous structure of $LiFeOCl_2$ to investigate the thermodynamic stability.

Reverse Monte Carlo modeling:

The supercell-based approach was used for total scattering data modeling. A conventional metropolis routine was used to adjust the model to the data in a reverse Monte Carlo (RMC) manner [11]. The RMC Profile software package was utilized for this purpose. The structure file calculated based on AIMD was used as the initial supercell for RMC modeling after cell expansion. The supercell has a size of 30.74 × 66.21 × 32.08 Å and a total of about 3512 atoms.

Finite element simulation:

Finite element simulations were performed to calculate the concentration and stress distribution of composite cathodes during the discharge/lithiation process [12]. The upper boundary of the model is defined as the current boundary condition, which is consistent with the current during the discharge process. The distribution of the stresses and concentration were simulated using COMSOL Multiphysics 6.3 software. In the model, the concentrations of catholytes and cathode particles are described by equation 2, including diffusion and electromigration.

$$J= -D \nabla c-z\ u\ F\ c\ \nabla\varphi \qquad (2)$$

In the equation, J is the total flux of Li^+ transmission, D is the diffusion coefficient, c is the concentration of Li^+ to be solved, z is the charge number of the reactive ion, F is the

Faraday constant (96500 C mol^{-1}), φ is the electric potential, ∇ is the Laplace symbol ($\nabla = \frac{\partial}{\partial x} + \frac{\partial}{\partial y} + \frac{\partial}{\partial z}$).

Based on the Butler-Volmer equation (Equation 3), the local current density and the velocity of the Faraday reaction are calculated.

$$I_l = i_0 \left\{ \exp\left(\frac{\alpha F \eta}{RT}\right) - c_{int} \exp\left(\frac{\beta F \eta}{RT}\right) \right\} \quad (3)$$

In the equation, i_l is the local current density of CAM, i_0 is the exchange current density, α and β is the reaction exchange coefficient, η is the reaction overpotential.

Then, the coupling of the Faraday reaction to the Li^+ concentration is realized based on Equation 4.

$$I_l = F\ J \quad (4)$$

In the process of Li^+ intercalation, the cathode particles will swell, which is proportional to their Li^+ intercalation concentration. It can be described by Equation 5.

$$V \propto V_{(c)} \quad (5)$$

Finally, the stress is calculated by Equation 6, which enables the coupling and calculation of the concentration and internal stress.

$$\sigma = E \frac{V}{V_0} \quad (6)$$

E is the modulus of the material and V_0 is the initial volume.

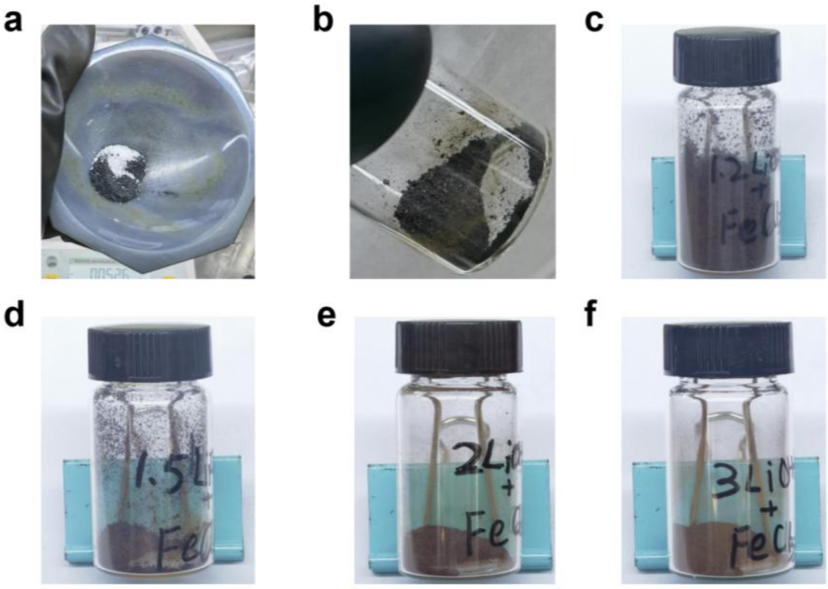

Figure S1. The optical images of (a) raw materials, (b) as-prepared materials, and (c-f) samples of xLiOH-$FeCl_3$ (x=1.2, 1.5, 2, 3).

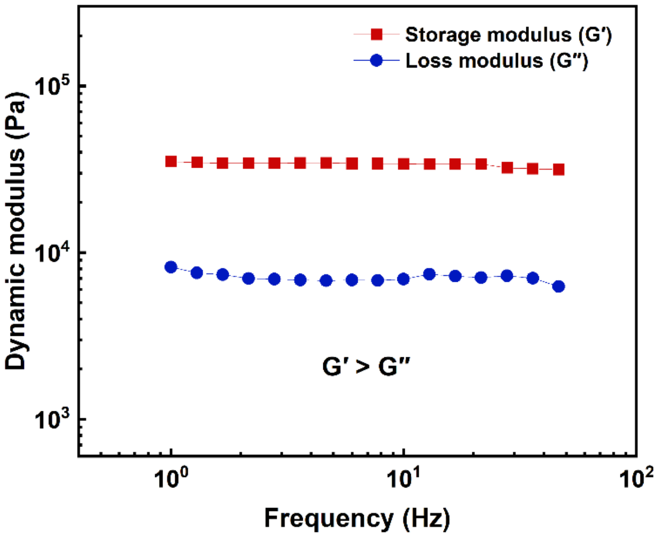

Figure S2. Rheological properties of the 1.2LiOH-$FeCl_3$ catholyte.

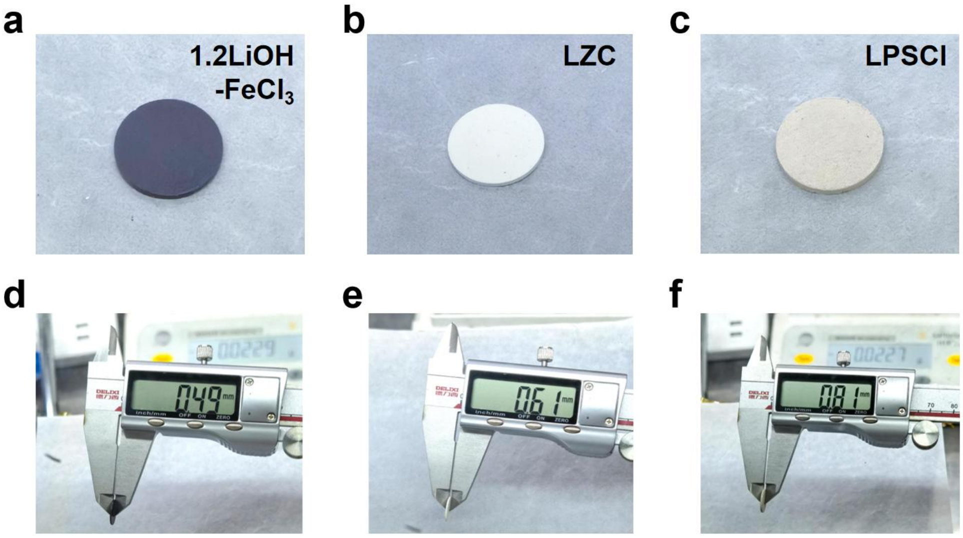

Figure S3. Optical images and corresponding thicknesses of 100 mg (a, d) 1.2LiOH-$FeCl_3$, (b, e) LZC, and (c, f) LPSCl cold-pressed pellets under 125 MPa.

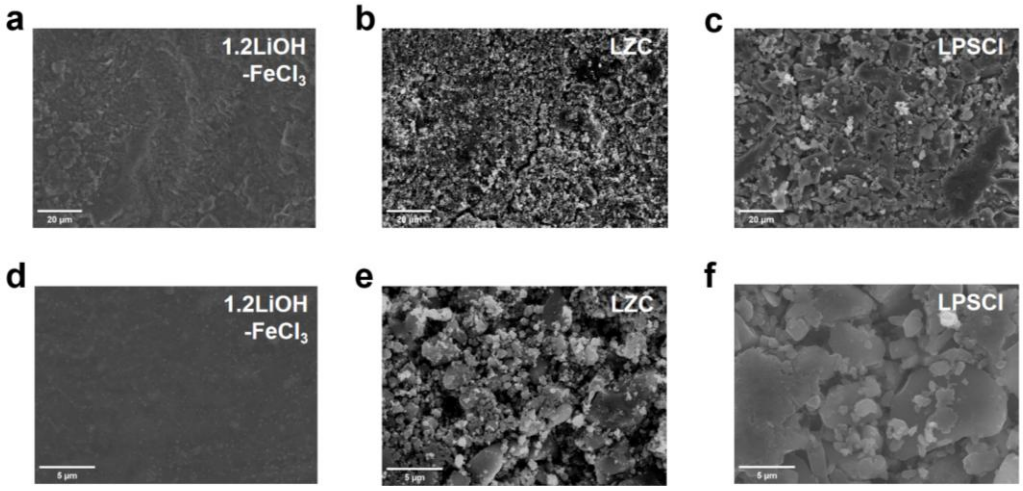

Figure S4. Surface and cross-sectional SEM images of (a, d) 1.2LiOH-$FeCl_3$, (b, e) LZC, and (c, f) LPSCl pellets under 125 MPa.

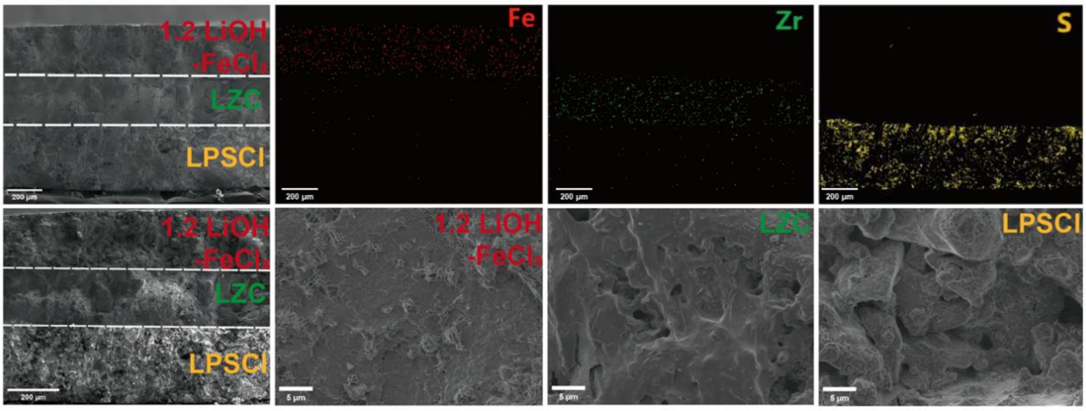

Figure S5. The cross-sectional SEM images and EDS mappings of the ASSLB configuration using 1.2LiOH-$FeCl_3$ CAM under 125 MPa.

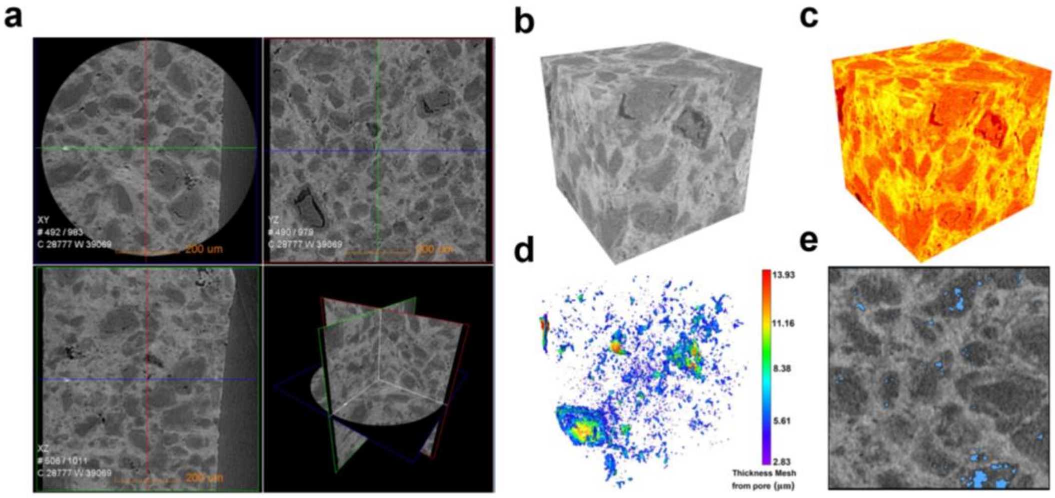

Figure S6. The XCT images of 1.2LiOH-$FeCl_3$ cold-pressed pellet at 125 MPa. (a) Reconstructed 2D slices were observed using ZEISS's 3D Viewer software. 3D rendered images from 0.7 μm voxel resolution scan of (b) grayscale 3D rendered image, (c) pseudo-color rendered image, and (d) mesh 3D model for pore thickness analysis. (e) Virtual slice gray map with 0.7μm voxel resolution using Dragonfly software.

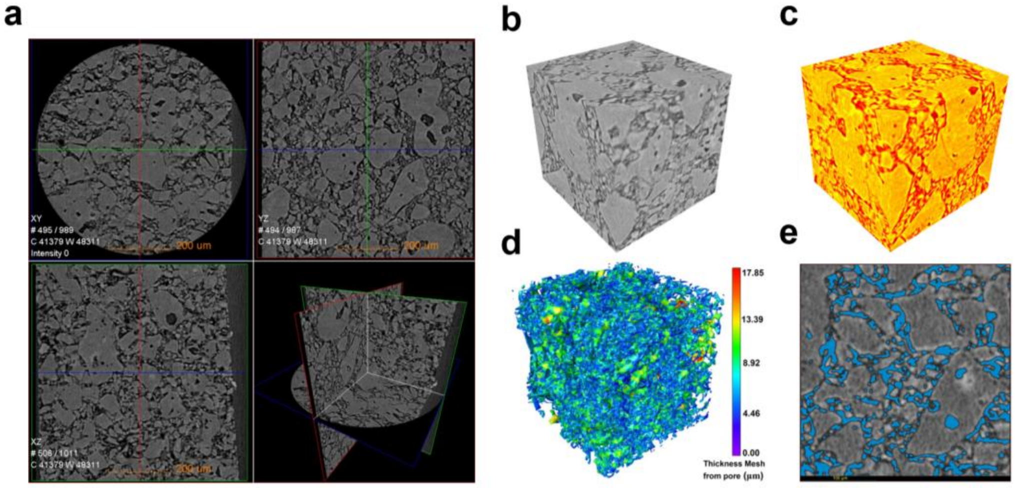

Figure S7. The XCT images of LPSCl cold-pressed pellet at 125 MPa. (a) Reconstructed 2D slices were observed using ZEISS's 3D Viewer software. 3D rendered images from 0.7 µm voxel resolution scan of (b) grayscale 3D rendered image, (c) pseudo-color rendered image, and (d) mesh 3D model for pore thickness analysis. (e) Virtual slice gray map with 0.7µm voxel resolution using Dragonfly software.

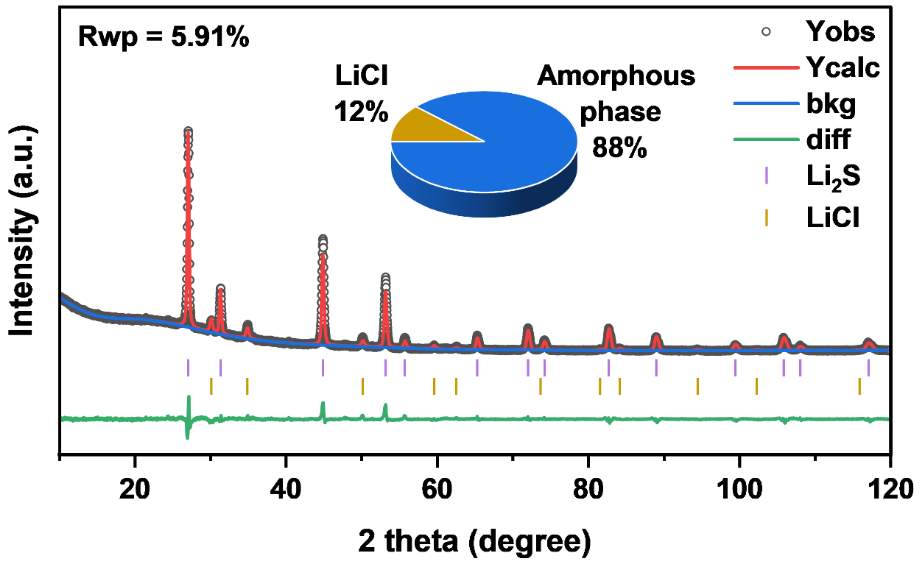

Figure S8. Quantification of the nanocrystalline LiCl content in the 1.2LiOH-$FeCl_3$ using internal standard method.

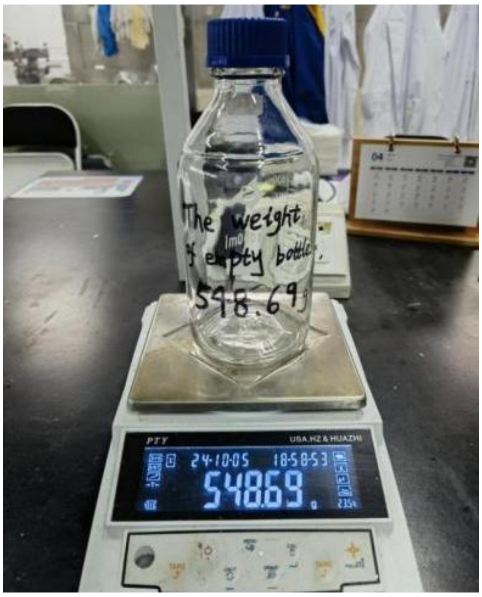

Figure S9. The weight of an empty bottle is 548.69 g.

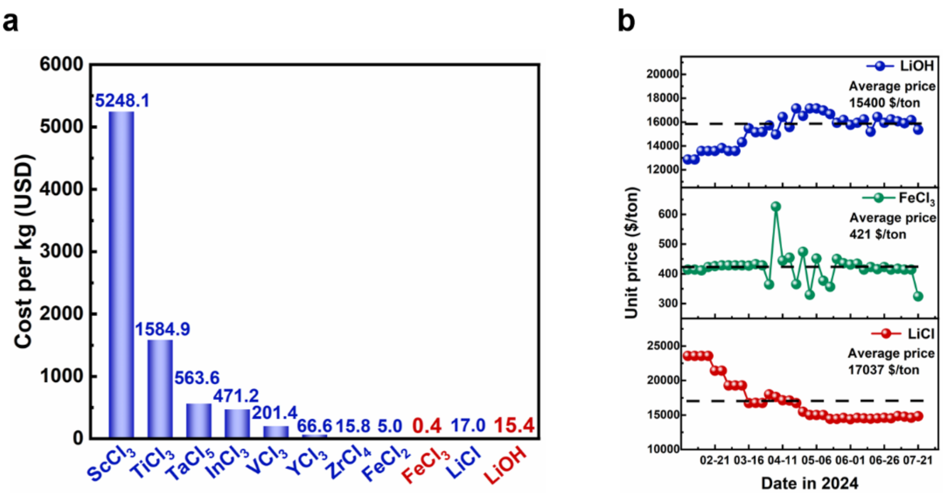

Figure S10. (a) Estimated unit prices of different raw materials and catholyte-free cathodes [4]. The price of commercial $FeCl_2$ is collected from Shanghai Epoch Material Co., Ltd. (b) The average prices of several commercial raw materials for catholyte-free cathodes in China. The data source is collected from https://m.chemicalbook.com/.

Note: The bulk prices plotted here are also listed in Fig. S9 and Table S1, and several laboratory-scale prices used to estimate these bulk prices are provided in Table S2. The price of LiOH is approximately \$15.4 kg^{-1}, comparable to the Li source of LiCl (\$17.0 kg^{-1}) for synthesizing Li_3ScCl_6, Li_3TiCl_6, Li_2TaCl_7, Li_3InCl_6, Li_3VCl_6, Li_3YCl_6, Li_2ZrCl_6, and Li_2FeCl_4. However, the price of $FeCl_3$ (\$0.4 kg^{-1}) is only 0.0076%, 0.03%, 0.071%, 0.085%, 0.6%, 2.5%, and 8% of estimated $ScCl_3$ (\$5248.1 kg^{-1}), $TiCl_3$ (\$1584.9 kg^{-1}), $TaCl_5$ (\$563.6 kg^{-1}), $InCl_3$ (\$471.2 kg^{-1}), VCl_3 (\$201.4 kg^{-1}), YCl_3 (\$66.6 kg^{-1}), $ZrCl_4$ (\$15.8 kg^{-1}), and $FeCl_2$ (\$5.0 kg^{-1}). As a result, the as-synthesized 1.2LiOH-$FeCl_3$ demonstrates an ultralow price of \$2.6 kg^{-1}, which is much lower than the reported Li_3ScCl_6 (\$2859.1 kg^{-1}), Li_3TiCl_6 (\$876.2 kg^{-1}), $LiTaOCl_4$ (\$508.2 kg^{-1}), Li_2TaCl_7 (\$459.0 kg^{-1}), Li_3InCl_6 (\$265.9 kg^{-1}), Li_3VCl_6 (\$119.0 kg^{-1}), Li_3YCl_6 (\$47.1 kg^{-1}), Li_2ZrCl_6 (\$16.1 kg^{-1}), and Li_2FeCl_4 (\$9.8 kg^{-1}).

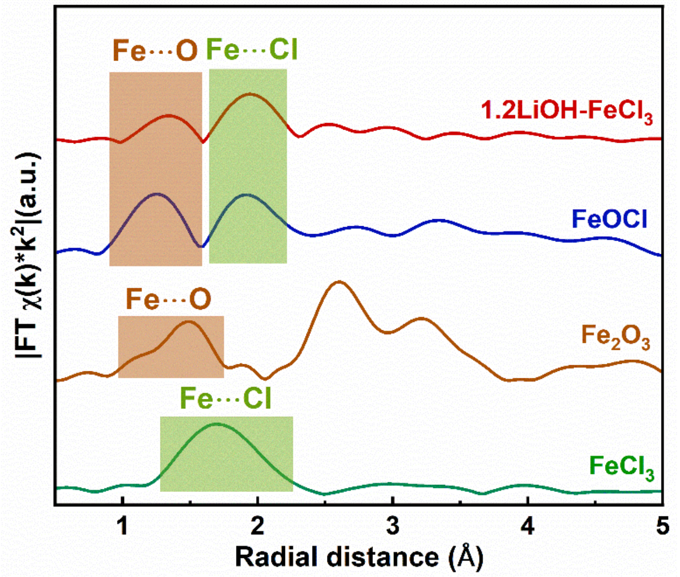

Figure S11. Fe-K edge R space EXAFS spectra of 1.2LiOH-$FeCl_3$, FeOCl, Fe_2O_3, and $FeCl_3$.

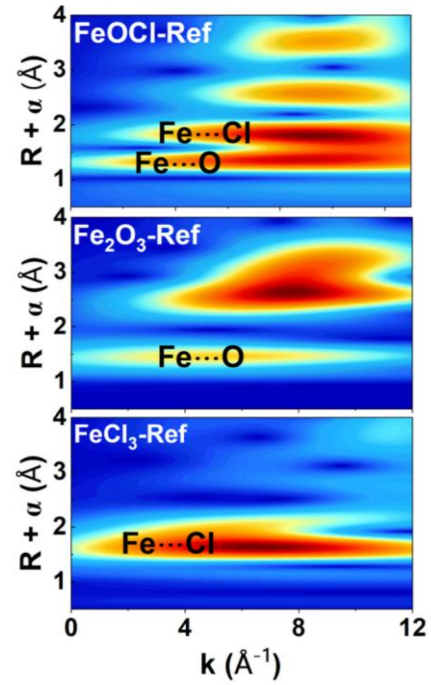

Figure S12. Wavelet transforms (WT) for the k^3-weighted Fe K-edge EXAFS signals of $FeCl_3$, Fe_2O_3, and FeOCl.

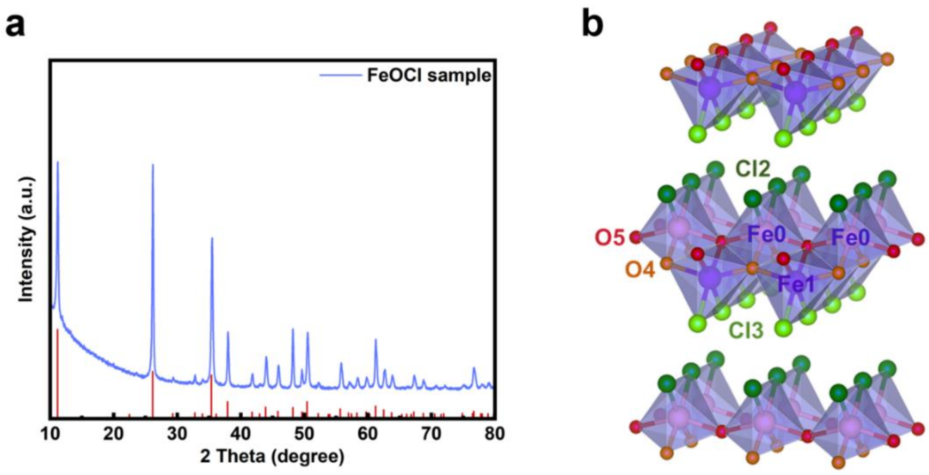

Figure S13. (a) The XRD pattern of the prepared FeOCl referential sample [14] and (b) Corresponding crystal structure diagram.

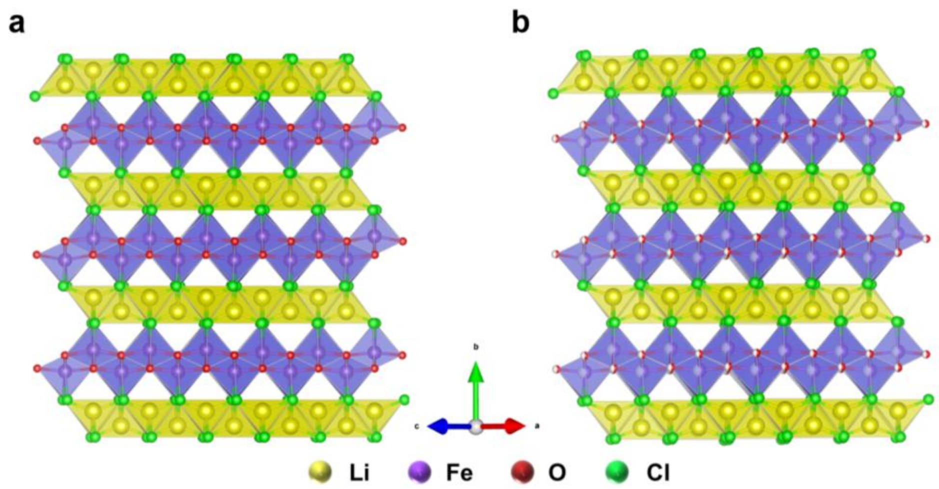

Figure S14. Crystal structures of (a) $LiFe_2O_2Cl_2$ and (b) $LiFeOCl_2$. (a) $LiFe_2O_2Cl_2$ adopts a monoclinic symmetry with a P2/c space group, featuring fully occupied Wyckoff positions 2e and 2f for lithium (Li) and iron (Fe), respectively, coordinated by oxygen (O) and chlorine (Cl) atoms at the 4g sites. This arrangement ensures robust ionic interactions and structural stability. (b) In $LiFeOCl_2$, the same monoclinic symmetry and space group is retained, but with vacancies introduced at the 2e, 2f, and 4g positions, leading to partial occupancy (0.5) for both Fe and O atoms.

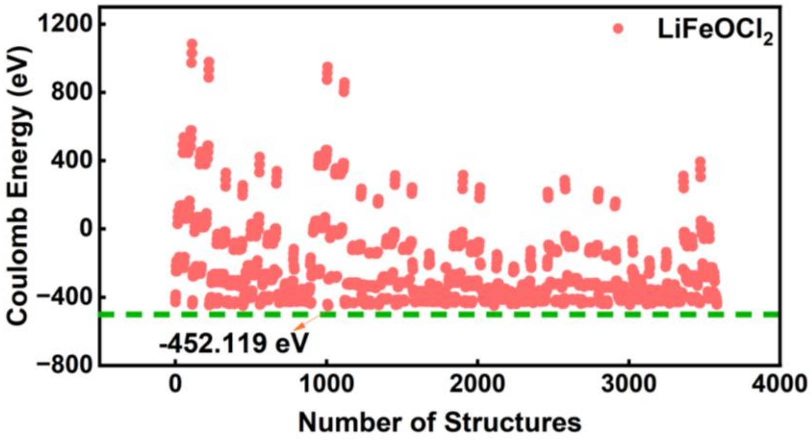

Figure S15. Structural configurations of $LiFeOCl_2$ generated using the Supercell program.

Note: The plot presents the Coulomb energy (in eV) versus various structural configurations, highlighting the identification of the most stable arrangement, which corresponds to the structure with the lowest Coulomb energy. The arrow indicates the configuration that minimizes electrostatic interactions within the material.

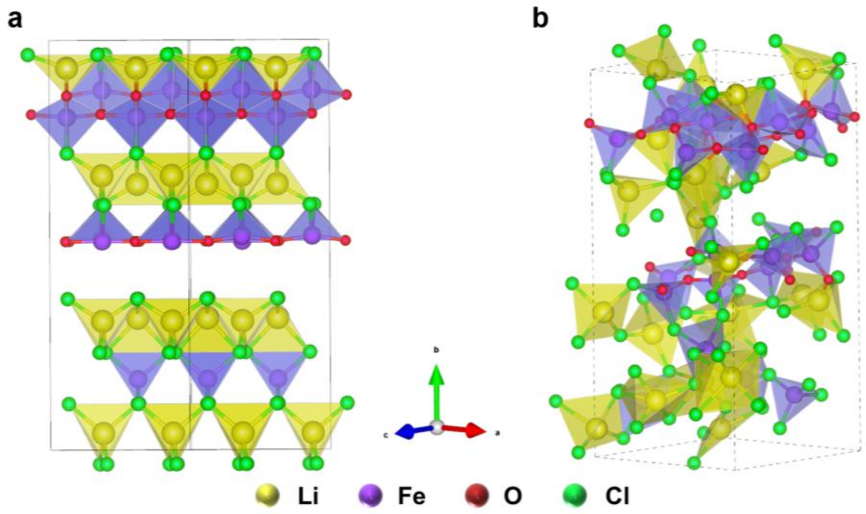

Figure S16. (a) Panel illustrates the optimized structure after DFT spin-polarized calculations, highlighting significant changes in atomic positions and bond lengths, resulting in a more stable configuration. (b) Panel depicts the melted structure obtained from AIMD simulations at 300 K, reflecting a disordered arrangement of iron-containing oxyhalide polyanions.

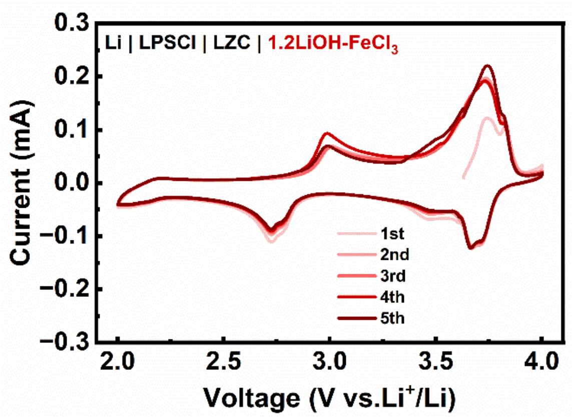

Figure S17. Cyclic voltammetry (CV) curves of the initial five cycles for the 1.2LiOH-$FeCl_3$ cathode at a sweep rate of 0.1 mV s^{-1}.

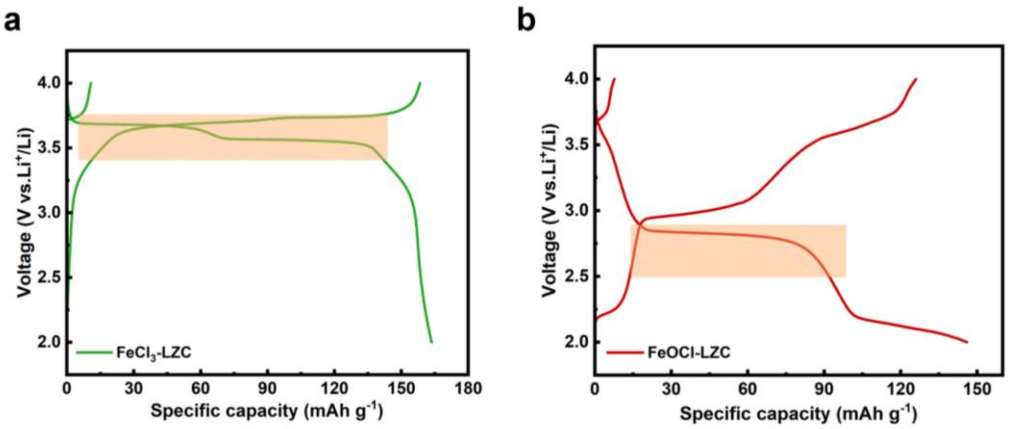

Figure S18. The discharge curves of (a) $FeCl_3$-Li_2ZrCl_6-KB and (b) FeOCl-Li_2ZrCl_6-KB cathodes at 0.1 mA cm^{-2}.

Note: For FeOCl-LZC ASSLBs, composite cathodes were prepared by ball milling FeOCl (synthesized by the CVD method) [13], LZC, and KB at a weight ratio of FeOCl/LZC/KB = 70/25/5 for two hours. For $FeCl_3$-LZC ASSLBs, composite cathodes are prepared by ball milling $FeCl_3$ (99.9%, anhydrous, Aladdin), LZC, and KB at a weight ratio of $FeCl_3$/LZC/KB = 50/45/5 at 180 r/min for two hours [14]. The same procedure as the 1.2LiOH-$FeCl_3$ cathode is applied for the ASSLB assembly.

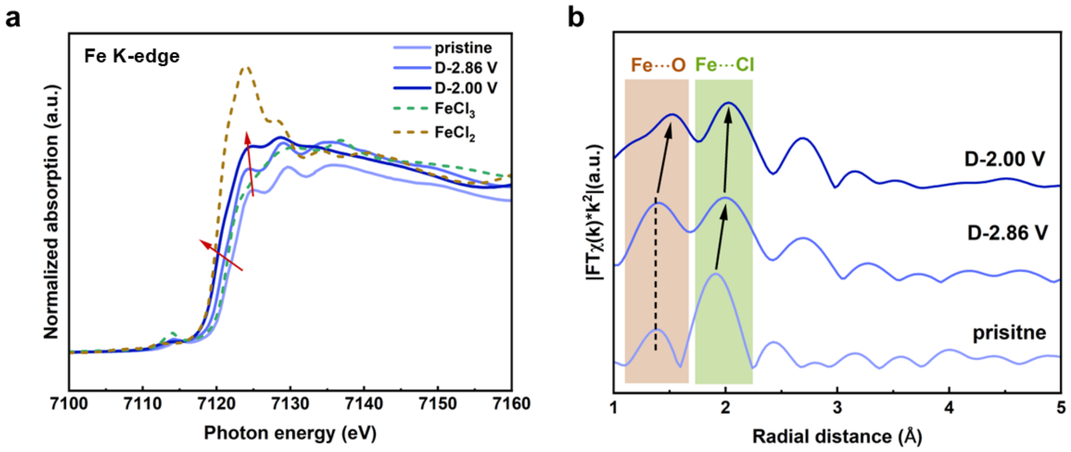

Figure S19. The ex situ XAS data of the 1.2LiOH-$FeCl_3$ cathode during the discharging process. (a) Ex situ Fe K-edge XANES spectra and (b) Fe K-edge R space EXAFS spectra of 1.2LiOH-$FeCl_3$ at different states of discharge.

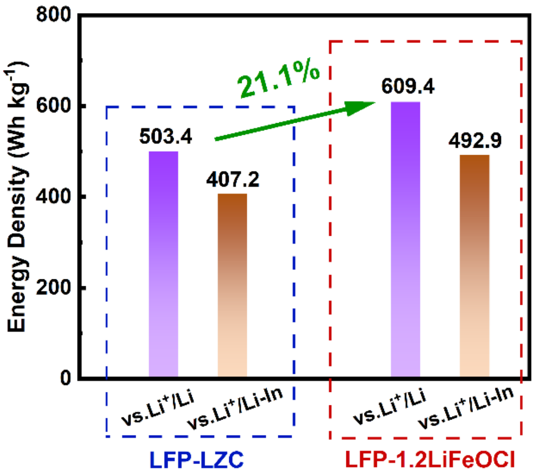

Figure S20. Comparison of the energy density of the LFP-LZC and LFP-1.2LiFeOCl composite cathodes.

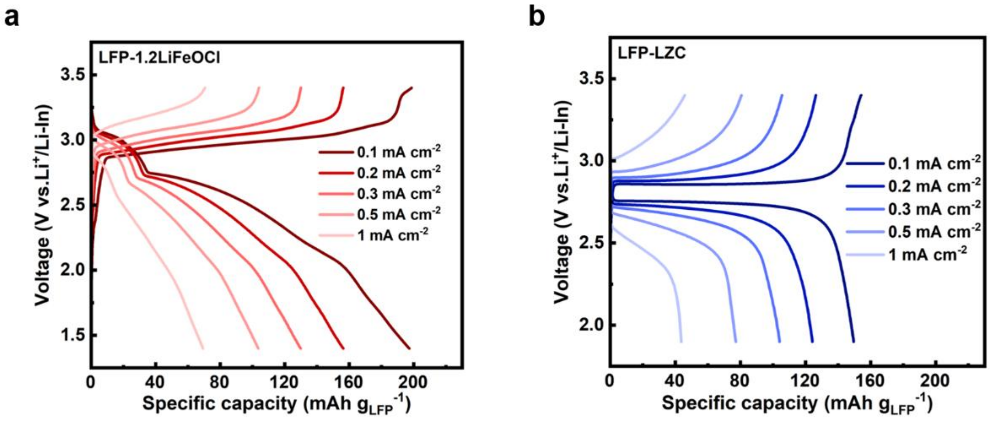

Figure S21. Rate performance of the zero-pressure ASSLB using (a) LFP-1.2LiFeOCl and (b) LFP-LZC composite cathodes.

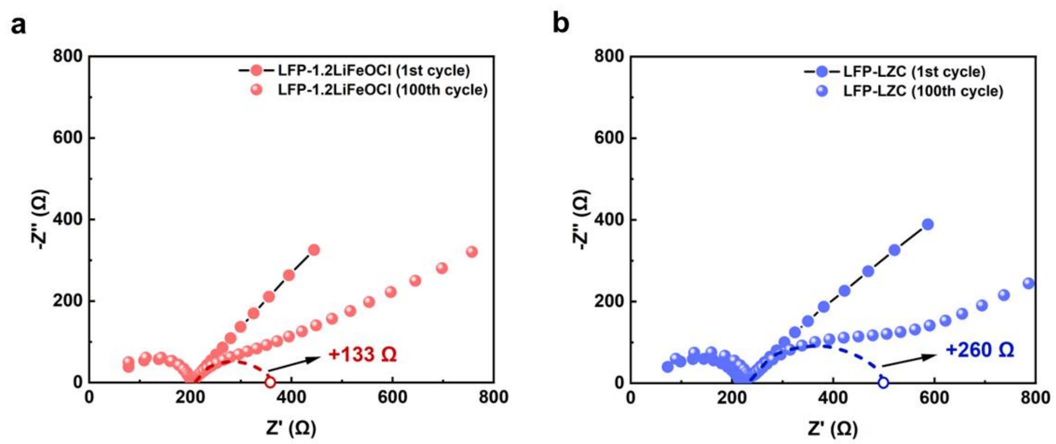

Figure S22. Impedance changes of (a) LFP-1.2LiFeOCl and (b) LFP-LZC zero-pressure ASSLBs.

Note: The LFP-1.2LiFeOCl ASSLB shows lower initial impedance (~200 Ω) than its LFP-LZC counterpart (~220 Ω) after the first cycle, due to the higher ionic conductivity of the 1.2LiOH-$FeCl_3$ catholyte. Following 100 cycles, a new semicircle emerges in the Nyquist plots of both zero-pressure ASSLBs, corresponding to charge transfer resistance at interfaces [17]. Given that catholyte is the sole difference between the cells, the smaller impedance growth in the LFP-1.2LiFeOCl ASSLB (+133 Ω vs. +260 Ω for LFP-LZC) underscores the superior interfacial stability imparted by the viscoelastic 1.2LiOH-$FeCl_3$ catholyte within the composite cathode during zero-pressure cycling.

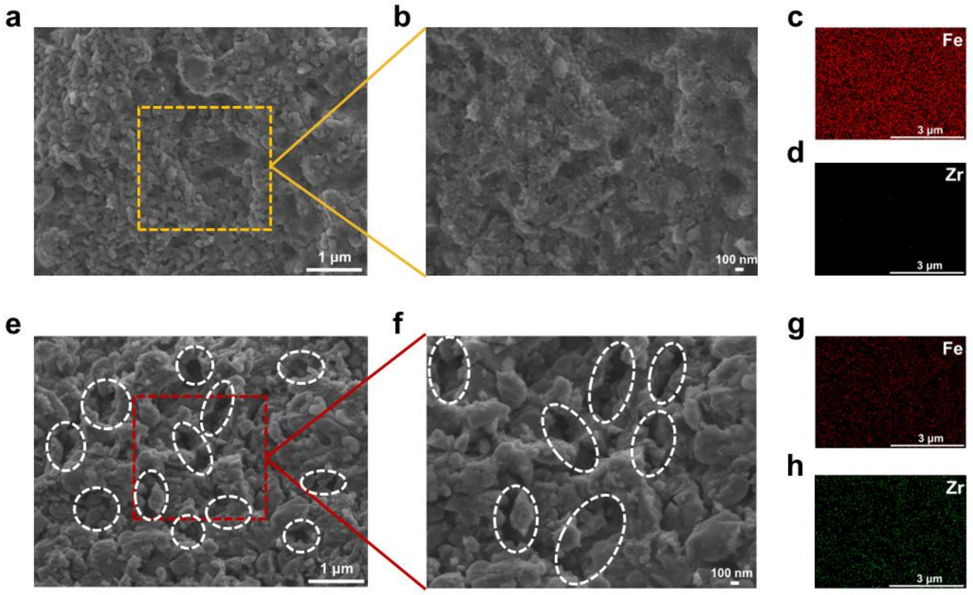

Figure S23. (a, b) The cross-sectional SEM images and (c, d) corresponding EDS mapping images of LFP-1.2LiFeOCl composite cathode after cycling. (e, f) The cross-sectional SEM images and (g, h) corresponding EDS mapping images of LFP-LZC composite cathode after cycling.

Note: The white circles represent voids and cracks.

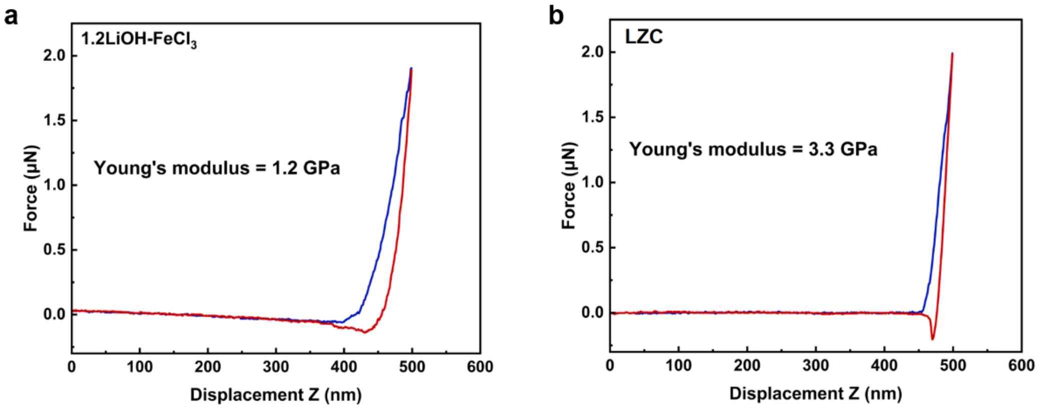

Figure S24. The Young's modulus of (a) 1.2LiOH-$FeCl_3$ and (b) LZC.

Note: The Young's modulus of 1.2LiOH-$FeCl_3$ and LZC are 1.2 GPa and 3.3 GPa, respectively.

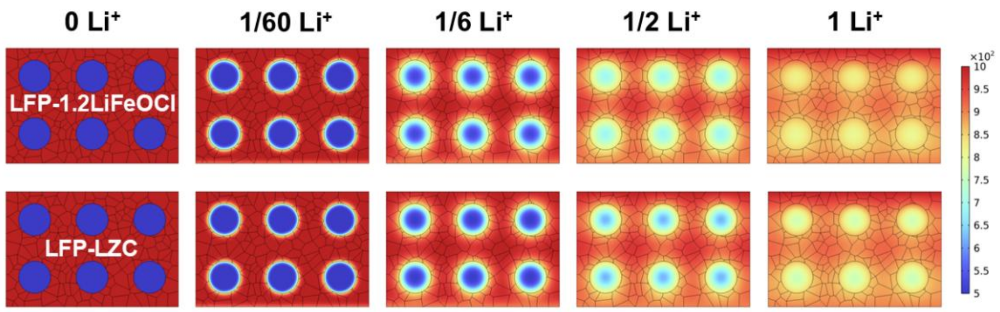

Figure S25. Dynamic evolution of Li^+ concentration for LFP-1.2LiFeOCl (top) and LFP-LZC (bottom) composite cathodes.

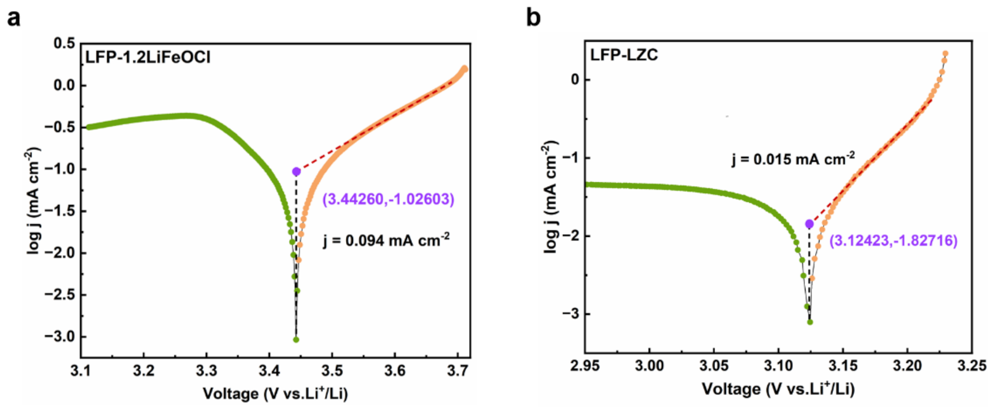

Figure S26. The exchange current densities of (a) LFP-1.2LiFeOCl (0.094 mA cm^{-2}) and (b) LFP-LZC (0.015 mA cm^{-2}), respectively.

Note: The higher exchange current density of the LFP-1.2LiFeOCl represents the faster electrochemical kinetic process.

Table S1. Comparison of the prices for raw materials needed to synthesize different types of cathodes. The bulk prices presented here are estimated from the laboratory-scale prices listed in Table S2 [4].

Raw materials	Intercept [log10(a)]	Slope[b]	Absolute Correl. Coeff. |r|	Unit price in bulk (1000 kg) purchase, \$ kg^{-1}
$TiCl_3$	3.7990	-0.1000	1	1584.9
VCl_3	4.2549	-0.2870	0.9483	201.4
$InCl_3$	3.6698	-0.1661	0.9500	471.2
YCl_3	3.6233	-0.3000	0.9815	66.6
$ZrCl_4$	3.3997	-0.3610	0.9905	15.8
$ScCl_3$	5.3950	-0.2686	1	5248.1
$TaCl_5$	4.0979	-0.2245	0.9760	563.6

Table S2. Laboratory-scale prices are used for the estimation in Table S1. The price of $TiCl_3$ listed here is taken from Alfa Aesar [3], and the price of VCl_3 listed here is taken from Adamas-beta [4]. The prices of anhydrous YCl_3, $InCl_3$, $ZrCl_4$, $ScCl_3$, and $TaCl_5$ listed here are taken from Aladdin.

Raw materials	Stock No.	Purchase quantity, Q, grams	Total purchase price, $	Unit price, P, $ kg^{-1}
$TiCl_3$ ($TiCl_3$-$AlCl_3$, 78.5%, Alfa - Aesar)	036729-22	78.5	322.7	4110.8
	036729-36	392.5	1361	3467.5
VCl_3 (97%, Adamas-bata)	82391A	1	22.3	22300
	82391B	5	52.7	10532
	82391C	25	188.4	7536
$InCl_3$ (99.99%, metal basis, Aladdin)	I196212-5g	5	19	3800
	I196212-25g	25	61.1	2444
	I196212-100g	100	224	2240
	I196212-500g	500	850.6	1701.2
YCl_3 (99.95%, metal basis, Aladdin)	Y119224-10g	10	21.3	2130
	Y119224-50g	50	60.6	1212
	Y119224-250g	250	210.1	840.4
$ZrCl_4$ (99.5%, metal basis, Aladdin)	Z109459-1g	5	8	1600
	Z109459-5g	25	18.7	748
	Z109459-25g	100	56	560
	Z109459-100g	500	162	324
$ScCl_3$ (99.9%, metal basis, Aladdin)	S137955-1g	1	248.5	248500
	S137955-5g	5	805	161000
$TaCl_5$ (99.8%, metal basis, Aladdin)	T301576-1g	1	12	12000
	T301576-5g	5	48	9600
	T301576-25g	25	143	5720
	T301576-100g	100	450	4500

Table S3. The crystallographic data of the FeOCl sample. The space group is P1. The lattice parameters are a = 3.27691 Å, b = 3.84811 Å, and c = 8.17802 Å. The unit cell volume is 103.123873 $Å^3$.

atoms	**x**	**y**	**z**	**Occ.**	**U**	**Site**	**Sym.**
Fe0	0.50000	0.00000	0.10925	1.000	0.000	1a	1
Fe1	0.00000	0.50000	0.89075	1.000	0.000	1a	1
Cl2	0.00000	0.00000	0.31957	1.000	0.000	1a	1
Cl3	0.50000	0.50000	0.68043	1.000	0.000	1a	1
O4	0.00000	0.00000	0.95296	1.000	0.000	1a	1
O5	0.50000	0.50000	0.04704	1.000	0.000	1a	1

Table S4. Summary of the bond length of the FeOCl sample.

bond	Fe0-Fe1	Fe0-O4	Fe1-O5	Fe0-Cl2	Fe1-Cl3
Bond length/Å	3.09505	2.07802	1.99017	2.37552	2.37552

Table S5. Detailed parameters of Finite element simulation.

LFP cathode	Ionic conductivity (25 °C)	5.0×10^{-5} S cm^{-1} [18]
	Young's modulus	47.3 GPa [19]
	Increased volume	6.77% [20]
1.2LiOH-$FeCl_3$ catholyte	Ionic conductivity (25 °C)	6.1×10^{-3} S cm^{-1}
	Young's modulus	1.2 GPa
	Exchange current density between LFP and 1.2LiFeOCl	0.094 mA cm^{-2}
LZC catholyte	Ionic conductivity (25 °C)	6.9×10^{-4} S cm^{-1}
	Young's modulus	3.3 GPa
	Exchange current density between LFP and LZC	0.015 mA cm^{-2}

REFERENCES

1. Wang K, Ren Q, Gu Z *et al.* A cost-effective and humidity-tolerant chloride solid electrolyte for lithium batteries. *Nat Commun* 2021; **12**: 4410.
2. Hart PW, Sommerfeld JT. Cost estimation of specialty chemicals from laboratory-scale prices. *Cost Engineering* 1997; **39**: 31.
3. Wang K, Gu Z, Xi Z *et al.* Li_3TiCl_6 as ionic conductive and compressible positive electrode active material for all-solid-state lithium-based batteries. *Nat Commun* 2023; **14**: 1396.
4. Song Z, Dai Y, Wang T *et al.* An active halide catholyte boosts the extra capacity for all-solid-state batteries. *Adv Mater* 2024; **36**: e2405277.
5. Dudarev SL, Botton GA, Savrasov SY *et al.* Electron-energy-loss spectra and the structural stability of nickel oxide: An LSDA+ U study. *Phys Rev B* 1998; **57**: 1505.
6. Kresse G, Furthmüller J. Efficient iterative schemes for ab initio total-energy calculations using a plane-wave basis set. *Phys Rev B* 1996; **54**: 11169-1186.
7. Kresse G, Furthmüller J. Efficiency of ab-initio total energy calculations for metals and semiconductors using a plane-wave basis set. *Comput Mater Sci* 1996; **6**: 15-50.
8. Jain A, Ong SP, Hautier G *et al.* Commentary: The Materials Project: A materials genome approach to accelerating materials innovation. *APL Mater* 2013; **1**: 011002.
9. Monkhorst HJ, Pack JD. Special points for Brillouin-zone integrations. *Phys Rev B* 1976; **13**: 5188.
10. Blöchl PE. Projector augmented-wave method. *Phys Rev B* 1994; **50**: 17953.
11. Xu X, Liu Y, Kapitanova OO *et al.* Electro–chemo–mechanical failure of solid electrolytes induced by growth of internal lithium filaments. *Adv Mater* 2022; **34**: 2207232.
12. Yang Q, Cai J, Li G *et al.* Chlorine bridge bond-enabled binuclear copper complex for electrocatalyzing lithium–sulfur reactions. *Nat Commun* 2024; **15**: 3231.
13. Wang J, Hou K-P, Wen Y *et al.* Interlayer structure manipulation of iron oxychloride by potassium cation intercalation to steer H_2O_2 activation pathway. *J Am Chem Soc* 2022; **144**: 4294-299.
14. Liu Z, Liu J, Zhao S *et al.* Low-cost iron trichloride cathode for all-solid-state lithium-ion batteries. *Nat Sustain* 2024; **7**: 1492-500.
15. Zhou X, Jiang M, Duan Y, *et al*. Multi-electron transfer halide cathode materials based on intercalation-conversion reaction towards all-solid-state lithium batteries. *Angew Chem Int Ed* 2025, **137**: e202416635.
16. Fu J, Wang C, Wang S, *et al*. A cost-effective all-in-one halide material for all-solid-state batteries. *Nature* 2025: 1-8.
17. Duan Y, Hussain F, Liu H *et al*. Breaking the conductivity-capacity trade-off in MCl6 anionic framework: amorphous oxyhalide cathode materials enable ~ 1100 Wh kg^{-1} at cathode-level in all-solid-state lithium batteries. *Adv Mater* 2025: e13544.
18. Wang C, Hong J. Ionic/electronic conducting characteristics of $LiFePO_4$ cathode materials: The determining factors for high rate performance. *Electrochem Solid-*

State Lett 2007; **10**: A65.

19. Sedlatschek T, Krämer M, Gibson JSKL *et al.* Mechanical properties of heterogeneous, porous $LiFePO_4$ cathodes obtained using statistical nanoindentation and micromechanical simulations. *J Power Sources* 2022; **539**: 231565.
20. Zhang W-J. Structure and performance of $LiFePO_4$ cathode materials: A review. *J Power Sources* 2011; **196**: 2962-970.
